# Deep learning on graphs for multi-omics classification of COPD

**DOI:** 10.1371/journal.pone.0284563

**Published:** 2023-04-21

**Authors:** Yonghua Zhuang, Fuyong Xing, Debashis Ghosh, Brian D. Hobbs, Craig P. Hersh, Farnoush Banaei-Kashani, Russell P. Bowler, Katerina Kechris

**Affiliations:** 1 Department of Biostatistics and Informatics, University of Colorado Anschutz Medical Campus, Aurora, CO, United States of America; 2 Biostatistics Shared Resource, University of Colorado Cancer Center, University of Colorado Anschutz Medical Campus, Aurora, CO, United States of America; 3 Department of Pediatrics, School of Medicine, University of Colorado Anschutz Medical Campus, Aurora, CO, United States of America; 4 Channing Division of Network Medicine, Brigham and Women’s Hospital, Boston, MA, United States of America; 5 Division of Pulmonary and Critical Care Medicine, Brigham and Women’s Hospital, Boston, MA, United States of America; 6 Harvard Medical School, Boston, MA, United States of America; 7 Department of Computer Science and Engineering, University of Colorado Denver, Denver, CO, United States of America; 8 National Jewish Health, Denver, CO, United States of America; State University of New York at Oswego, UNITED STATES

## Abstract

Network approaches have successfully been used to help reveal complex mechanisms of diseases including Chronic Obstructive Pulmonary Disease (COPD). However despite recent advances, we remain limited in our ability to incorporate protein-protein interaction (PPI) network information with omics data for disease prediction. New deep learning methods including convolution Graph Neural Network (ConvGNN) has shown great potential for disease classification using transcriptomics data and known PPI networks from existing databases. In this study, we first reconstructed the COPD-associated PPI network through the AhGlasso (Augmented High-Dimensional Graphical Lasso Method) algorithm based on one independent transcriptomics dataset including COPD cases and controls. Then we extended the existing ConvGNN methods to successfully integrate COPD-associated PPI, proteomics, and transcriptomics data and developed a prediction model for COPD classification. This approach improves accuracy over several conventional classification methods and neural networks that do not incorporate network information. We also demonstrated that the updated COPD-associated network developed using AhGlasso further improves prediction accuracy. Although deep neural networks often achieve superior statistical power in classification compared to other methods, it can be very difficult to explain how the model, especially graph neural network(s), makes decisions on the given features and identifies the features that contribute the most to prediction generally and individually. To better explain how the spectral-based Graph Neural Network model(s) works, we applied one unified explainable machine learning method, SHapley Additive exPlanations (SHAP), and identified CXCL11, IL-2, CD48, KIR3DL2, TLR2, BMP10 and several other relevant COPD genes in subnetworks of the ConvGNN model for COPD prediction. Finally, Gene Ontology (GO) enrichment analysis identified glycosaminoglycan, heparin signaling, and carbohydrate derivative signaling pathways significantly enriched in the top important gene/proteins for COPD classifications.

## Introduction

Chronic obstructive pulmonary disease (COPD) is a common, genetically complex and clinically heterogenous disease with unclear pathogenesis [1]. Although prediction models for COPD have been trained on clinical features and imaging data, these features may not help reveal mechanisms contributing to the development of COPD [2–4]. For example, Macaulay *et al*. developed a model to predict COPD with health-claim data including age, sex, COPD-related resource utilization (such as oxygen use), and all-cause healthcare utilization. The final model correctly predicted COPD severity (Global Initiative for Chronic Obstructive Lung Disease (GOLD) guidelines) with 62.7% accuracy [3]. Although this model helps predict COPD status, the prediction accuracy could be biased since it uses COPD-related resource utilization features (such as oxygen use) to predict COPD status. In addition, Himes *et al*. developed a Bayesian network model to predict COPD in asthma patients with Electronic Medical Records including age, sex, COPD-related symptoms (short of breath), and diseases (bronchitis and bronchiolitis) [2]. Although the final model in Himes *et al*. study correctly predicted COPD with 83% accuracy computed as the area under the Receiver Operating Characteristic curve (AUROC), the prediction model was trained on the unbalanced dataset (46 cases vs 946 controls). In addition, the significant age difference between controls and cases in their study led to the finding that the Age variable by itself can achieve 81% accuracy, which questions the generalizability of the model to other data sets. In addition, Schroeder *et al*. recently developed a CNN imaging-based model, with physiologic lung function data (PFTs) for COPD prediction, which achieved 74.9% accuracy [5]. Although the CNN imaging model achieved good accuracy, it does not give insight into the molecular mechanism of COPD development.

With the rapid development of genomic and proteomic technologies, high-throughput omics data allow comprehensive characterization of complex diseases, empower disease-specific network construction, and provide potential biomarkers to predict complex heterogeneous diseases [6, 7]. The direct and indirect interactions of biological entities including mRNAs and proteins can be represented as regulatory network(s). Networks provide a graphical representation of molecular interactions that helps explain pathogenesis for other complex diseases such as COPD [8–10]. Li *et al*. integrated nine omics data blocks by similarity network fusion (SNF) and demonstrated the improvement of group clustering and classification through network-based multi-omics integration. However, this study included only 52 female subjects and did not investigate what features are important for group clustering and COPD classification. [11].

Recently graph-based neural network (GNN) techniques have emerged, providing an opportunity to leverage biological network information. Computational efficiency used to be a bottleneck for GNN. One method proposed by Defferrard *et al*. leverages the spectral Convolutional graph Neural Network (ConvGNN) and improves computational efficiency significantly by using the Chebyshev approximation technique [12]. As a result, this cutting-edge method outperforms the existing approaches in terms of accuracy in many experiments [13–15]. Based on the spectral-based ConvGNN, Rhee *et al*. developed a hybrid approach of relation network and localized Graph Convolutional Filtering to incorporate protein-protein interaction information and gene expression data (single omics data) for breast cancer subtype classification and achieved significantly better performance than many existing methods [15]. Rhee *et al*.’s study demonstrates that network topology-based methods exhibit superior statistical power in the classification of diseases as well as other network-based methods including Paradigm and NetGSA [16, 17]. Schulte-Sasse *et al*. recently integrated mutations, copy number changes, DNA methylation, and gene expression together with protein-protein interaction (PPI) networks with graph convolutional networks for cancer prediction [18]. However, the use and interpretation of ConvGNN for integrating multi-omics data are still not well developed for complex diseases including COPD. In addition, Li *et al*. recently developed a novel method based on graph convolution network (GCN) for early detection of COPD with chest CT data and achieved higher accuracy than previous studies. However, it does not help reveal the molecular mechanism of COPD development [19].

In this study, we report a novel implementation of spectral-based ConvGNN for COPD classification: integrating multi-omics data (proteomics or transcriptomics) and disease-specific protein/genetic interaction graph information in the form of protein-protein interaction (PPI) networks. We demonstrate that ConvGNN outperforms many existing classification methods, such as Random Forests [20], Support Vector Machine (SVM) [21], and Scalable Tree Boosting System method (XGBoost) [22], which do not incorporate network information. We then extended the current ConvGNN to successfully incorporate two omics data (both transcriptomics and proteomics data) and achieved better performance than single omics data for COPD classification. Network information was retrieved from the STRING (Search Tool for the Retrieval of Interacting Genes/Proteins) database, one of the most comprehensive protein/genetic interaction databases [23]. However, PPI information specific to COPD in STRING might be limited. To build a more complete and specific protein-protein interaction network related to COPD, we used our previously developed AhGlasso (Augmented High-Dimensional Graphical Lasso Method) method [24] to reconstruct a network, and demonstrated how integrating the updated COPD-associated PPI further improves performance. To evaluate stability of the ConvGNN model, we used cross-validation to measure classification performance. Finally, to interpret the ConvGNN model, we applied SHapley Additive exPlanations (SHAP) analysis values [25] to identify top significant genes/proteins and important subnetworks for COPD.

## Materials and methods

### COPDGene study population and ethics statement

The NIH-sponsored multicenter Genetic Epidemiology of COPD (COPDGene, ClinicalTrials.gov Identifier: NCT00608764) study was approved and reviewed by the institutional review board at all participating clinical centers [26–28]. All study participants provided written informed consent. COPDGene study enrolled 10,198 participants with and without Chronic Obstructive Pulmonary Disease (COPD) between 2007 and 2011 (Phase I study) to identify genetic factors associated with COPD [27]. Participants were brought back in a five-year follow-up visit from 2013 to 2017 (Phase II study). Each in-person visit included spirometry before and after albuterol, quantitative computed tomography (CT) imaging of the chest, and blood sampling. In this study, we focus on -omics data sets collected in Phase 2 based on blood sampling. Data analyzed in this work were de-identified.

### Clinical variables and definitions

COPD was defined by post-bronchodilator spirometry FEV1/FVC < 0.7, where FEV1 is forced expiratory volume in the first second and FVC is Forced Vital Capacity. Subjects who did not fall into these categories were defined as controls. The Global Obstructive Lung Disease (GOLD) system was used to grade the severity of airflow limitation: mild—GOLD 1 (FEV1 ≥ 80%), moderate—GOLD 2 (50% ≤ FEV1 < 80%), severe—GOLD 3 (30% ≤ FEV1 < 50%), and very severe—GOLD 4 (FEV1 < 30%) [29]. Of note, GOLD 0 represents at-risk stage and was defined by the presence of risk factors (smoking) and symptoms (chronic cough and sputum production) in the absence of post-bronchodilator spirometry abnormalities that cross the diagnostic threshold for COPD (FEV1/FVC < 0.7) [30]. In other words, GOLD 0 represents an individual at-risk stage (chronic smoker) but without COPD (control).

### Proteomics data and processing

Proteomic profiles were constructed on participants who agreed to participate in the ancillary study of COPDGene Phase II. All analyses were performed on frozen plasma from p100 tubes as previously described [31]. 1086 subjects passed QC in proteomic profiling. The never smoker controls and subjects who had lung transplants before Phase II were excluded.

For the SOMAscan 1.3k assay, intra-run normalization and inter-run calibration were performed according to SOMAscan assay data quality-control procedures as defined in the SomaLogic good laboratory practice quality system. Data from all samples passed recommended quality-control criteria by SOMAscan (Sample Normalization 0.4—2.5, Plate Scale Factor 0.4—2.5, SOMAmer Calibration median ± 0.4, Plate Median Scale Factor 0.8—1.2, Plate tail test less than 10%) and were fit for analysis. The data were standardized to have mean 0 and standard deviation 1. To map with the STRING database, the proteomics expression data without one-to-one mapping to gene symbols were removed. For example, if two SomaScan® aptamers map to the same gene symbol, these two aptamers’ corresponding expression data were removed. In addition, some aptamers either detect the expression level of a protein complex or detect the total expression level of several proteins by targeting a shared subunit. These aptamers were removed as well for simplicity. Expression data for 1212 proteins were retained for analysis.

### Transcriptomics data and processing

Transcriptomic profiles were also generated to identify genomic factors associated with COPD [9, 27, 32] at Phase II. The mRNA high-throughput sequencing data was generated from peripheral blood samples collected and previously described [33]. Briefly, total RNA was extracted from PAXgene ™ Blood RNA tubes and cDNA library preparation was performed with the Illumina TruSeq Stranded Total RNA kit (Illumina, Inc., San Diego, CA). 75 bp paired-end reads were generated on Illumina sequencers. Reads were trimmed of TruSeq adapters and then aligned to the GRCH38 genome. Quality control was performed using the FastQC and RNA-SeQC programs [34, 35].

The complete CODPGene total mRNA sequencing data contained 60232 transcripts that were measured on 3985 peripheral blood samples. For the purposes of this study, we filtered down to the 19889 protein-coding genes through Ensemble annotation by “biomaRt” R package [36]. We then applied upper-quartile normalization and Remove Unwanted Variation Using Residuals (RUVr) [37] to remove unwanted variance including batch effect. The generalized linear model used in RUVr to determine residuals includes the following covariates: sex, race, age, and GOLD stage. RUV utilizes the residuals from a first-pass generalized linear model (GLM) regression of the counts on the covariates. The “k” for RUVr was set to 3 because the variation decreased drastically after adding the first three components. Finally, the corrected sequencing counts were transformed to be homoscedastic via a variance stabilizing transformation (VST) [38].

### Proteomic-transcriptomic integration

719 subjects were profiled using both proteomics and transcriptomics. Because there is clinical heterogeneity of COPD across all stages of airflow limitation, we filtered out the subjects with GOLD = 1 since its diagnosis might be problematic per discussion with clinical experts. The moderate-to-severe (GOLD 2–4) subjects were used to represent cases since they are a more homogenous group of COPD as has been the standard for COPD genetic studies [39]. In total there were 614 subjects including 296 controls and 318 COPD cases (GOLD 2–4).

Among the above 1212 SomaScan® detected proteins, 1183 of them had corresponding mRNA transcript measurements. We focused on these 1183 pairs of mRNAs/proteins to construct the ConvGNN model for multi-omics integration. Of note, 25 pseudo nodes with neutral value (0) were added to the input matrix to ensure fast pooling of graph signals in graph neural network model training.

### STRING PPI database

STRING (http://www.STRING-db.org) is a database of known and predicted protein-protein interactions [23]. STRING currently covers 5,214,234 proteins from 1133 organisms. In STRING, protein-protein pair associations (i.e., the “edge weights” in each network) are represented by confidence scores. The scores indicate the estimated probability that a given interaction is biologically meaningful, specific, and reproducible, given the supporting evidence. There are seven evidence channels in STRING: (1) experiments; (2) database; (3) text-mining; (4) coexpression; (5) neighborhood; (6) fusion; and (7) co-occurrence. The edge scores (weights) between proteins in the STRING PPI database range from 0 to 1, with 1 being the highest possible confidence of interaction [23]. The edge score is the probability that the protein-protein interaction really exists given the available evidence [23]. We retrieved human PPI data from STRING and filtered out edges with scores less than 0.4, the default threshold in “STRINGdb” R package to achieve medium confidence to well balance false positive and false negative as recommended by STRING [23, 40].

### PPI reconstruction with AhGlasso

Using the prior PPI network retrieved from the STRING database, we applied our previously developed AhGlasso algorithm [24] on the transcriptomics data of 2656 subjects without proteomics measurements but with known clinical phenotype data to construct COPD-associated networks. In order to find the optimal regularization parameter, λ, networks were estimated under a sequence of λ values. The upper bound λ_*max*_ was calculated as described previously [24]. With predefined 0.01 λ minimal ratio, λ_*min*_ = 0.01 * λ_*max*_. Then 40 λ candidates were generated between λ_*min*_ and λ_*max*_ in a log scale for grid search. The optimal λ was selected using the Bayesian Information Criterion (BIC) based on the penalized log Likelihood with 4-fold cross-validation and one standard error rule [24]. The resultant network serves as the updated COPD-associated PPI network for model development.

### Localized pattern representation by Convolutional Graph Neural Network

ConvGNNs have recently become a popular approach for graph data because of their ability to extract features from graphs [13, 15, 41]. ConvGNN approaches are divided into two categories, spatial-based and spectral-based. The spatial-based ConvGNN methods are often based on random walk and mainly used for node prediction. The spectral-based methods are built based on spectral graph theory and useful for graph prediction. In this study, we are interested in COPD disease prediction (graph prediction) but not individual gene/protein function (node prediction). Therefore, we focus on the spectral-based ConvGNN development and compare its performance with existing classification methods. As described in Rhee et al.’s study [15], for capturing localized patterns of data (gene/protein expression profile), the input data is mapped onto the graph structure with the graph convolution technique.

Let expression data be ***X***_*n*×*p*_, where *l* = 1, …, *n* denotes the number of samples and *i* = 1, …, *p* denotes the number of nodes (genes or proteins). Let *x*_*l*_ ∈ *R*_*n*_ be the vector of expression values of *p* genes/proteins in the sample *l*. Then the graph Laplacian matrix *L* is used to find the spectral localized patterns of *x*_*l*_ under the graph structure *G*. The Laplacian matrix *L*_*p*×*p*_ of graph *G* is defined as *L* = *D* − *A*, where *D* is a weighted degree matrix and *A* is the adjacency matrix of the graph. Then the graph convolution is defined with the graph Laplacian matrix. Let’s assume that *L* = *U*Λ*U*^*T*^ is an eigenvalue decomposition of graph Laplacian *L*, where *U* = [*u*_1_, …, *u*_*p*_] is a matrix composed of eigenvectors ${\left\{u_{i}\right\}}_{i=1}^{p}$ and Λ is a diagonal matrix *diag*[*τ*_1_, …, *τ*_*p*_] composed of eigenvalues ${\left\{\tau_{i}\right\}}_{i=1}^{p}$. Then the graph Fourier transform (*F*) is defined as $\hat{x}=F\left(x\right)=U^{T}x$ and the inverse graph Fourier transform (*F*^−1^) is defined as $x=F^{-1}\left(\hat{x}\right)=U\hat{x}$ [12, 42, 43].

Because it is hard to express a meaningful convolution operator in the vertex domain, Defferrard *et al*. defined the convolution operator on graph *_g_ in the Fourier domain as follows [12],

$$\begin{array}{c} \begin{array}{c} \underset{p\times 1}{y_{l}}=\underset{p\times p}{U}\underset{p\times p}{\hat{w}\left(\Lambda \right)}\underset{p\times p}{U^{T}}\underset{p\times 1}{x_{l}} \end{array} \end{array}$$ (1)

where *x*_*l*_ is the feature signal of nodes (*e.g*., gene/protein expression value in the first layer) in subject *l* while *y*_*l*_ is the transformed signal after graph convolution of subject *l*. The gene/protein expression data serve as signals of graph nodes in the PPI network and are convoluted with the graph convolution layers. Let the filter $\hat{w}=g_{\theta }$ with the convolution operator on graph *_g_ in Eq 1; we can extract high level feature (*y*) by filtering signal *x* with the filter *g*_*θ*_ as Defferrard *et al*.’s study [12],

$$\begin{array}{c} \begin{array}{c} y=Ug_{\theta }\left(\Lambda \right)U^{T}x=g_{\theta }\left(U\Lambda U^{T}\right)x=g_{\theta }\left(L\right)x \end{array} \end{array}$$ (2)

where *g*_*θ*_(Λ) is a diagonal matrix $diag(\left[\hat{g_{\theta }}\left(\tau_{1}\right),...,\hat{g_{\theta }}\left(\tau_{p}\right)\right])$, and the parameter *θ* is a vector of Fourier coefficients.

One graph convolution filter is a polynomial parameterized filter, $g_{\theta }\left(\Lambda \right)=\Sigma_{k=0}^{K-1}\theta_{k}\Lambda^{k}$ that can express localized patterns in *K*-hop neighboring nodes [12, 42]. However, learning polynomial parameters is computationally intensive $O(n^{2})$. In 2011, Hammond *et al*. proposed an approximated polynomial named Chebyshev expansion [42] to significantly reduce the computation burden and improve efficiency. The Chebyshev polynomial *T*_*k*_(*x*) of order *k* is recursively computed by the stable recurrence relation, *T*_*k*_(*x*) = 2*xT*_*k*−1_(*x*)−*T*_*k*−2_(*x*), where *T*_0_ = 1 and *T*_1_ = *x*. Thus, the filter can be approximated as $g_{\theta^{\prime }}\left(\Lambda \right)=\Sigma_{k=0}^{K-1}\theta_{k}^{\prime }T_{k}\tilde{\Lambda }$, where $\tilde{\Lambda }=2\Lambda /\left(\lambda_{max}-I_{n}\right)$. With the Chebyshev approximation technique, the entire filtering operation reduces to O(K|E|), where *E* is a set of edges in a PPI network [12, 15].

In spectral-based ConvGNN, the convolutional graph signal is further pooled with neighboring nodes identified by the Graclus algorithm (Pooling) as described previously [12, 15]. For fast pooling of graph signals, pseudo nodes with neutral value (0) are introduced in the graph neural network as previous study [12]. There are several pooling strategies in the neural networks such as max pooling and average pooling [44]. The average pooling was used in this study as discussed below.

### Multi-omics integration through ConvGNN for COPD classification

**Fig 1:**
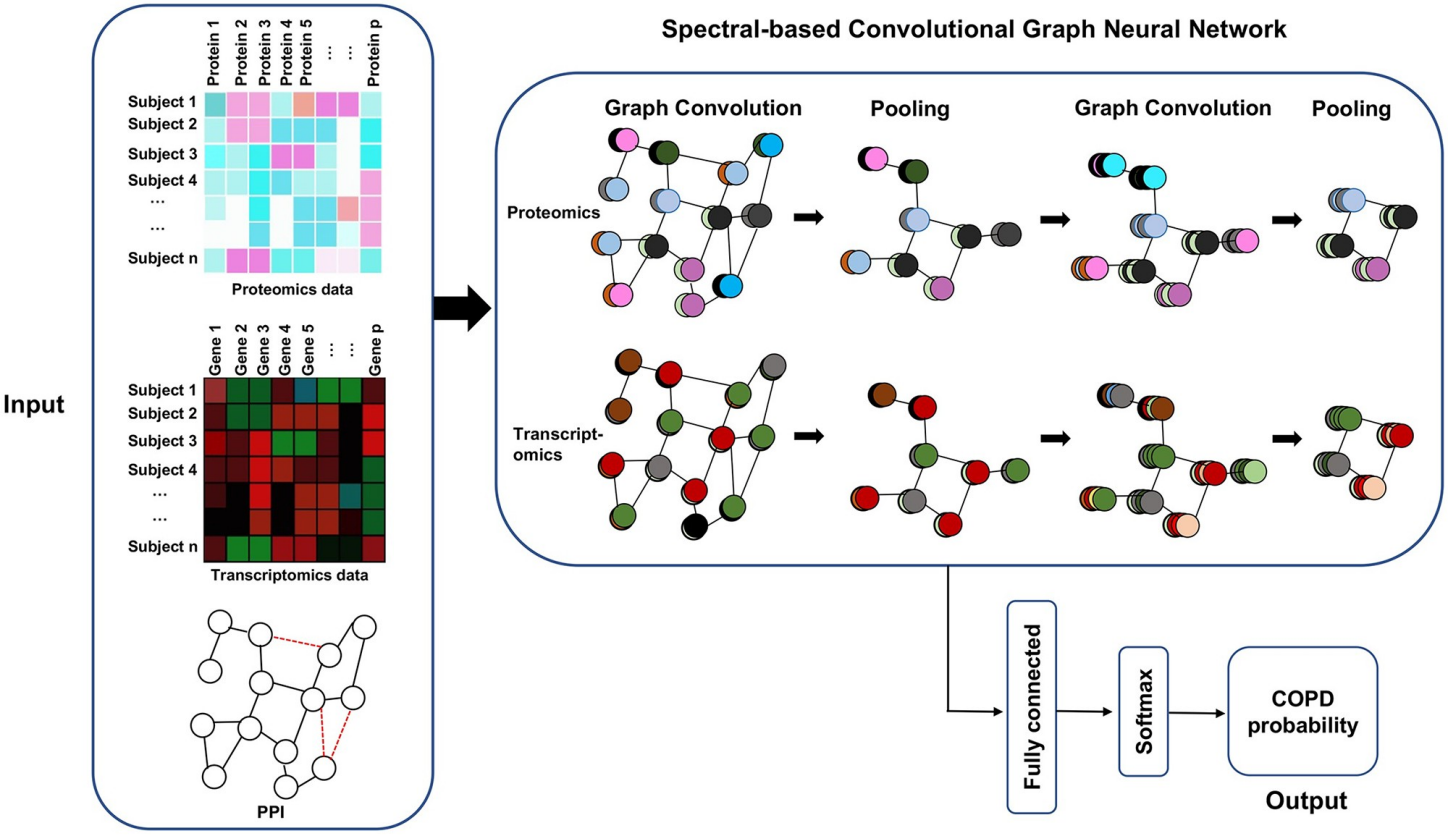
Overview of spectral-based Convolutional Graph Neural Network (ConvGNN) for COPD classification with single or multi-omics data. The inputs are a protein-protein interaction network (PPI) and omics data which could be single omics data only or multi-omics data. The PPI network could be retrieved from STRING databases or reconstructed from the AhGlasso algorithm. The red edges between nodes represent changes between the original PPI from STRING and the updated PPI with AhGlasso. The input is fed into a Spectral-based Convolutional Graph Neural Network, which typically includes layers for graph convolution and pooling to extract features with different kernels. The graph convolution and pooling could be repeated as shown on the top right. The resultant features will be passed to fully connected layers to calculate the probability of COPD using the softmax function.

The ConvGNN model developed in Rhee *et al*.’s study deals with single-omic data. We extend this model to integrate multi-omics data (transcriptomics and proteomics) with the protein-protein interaction (PPI) network in the ConvGNN (Fig 1) to test whether it could further improve COPD classification accuracy. We set the same convolution kernel for proteomics and transcriptomics data to reduce the computation burden. The extracted features through graph convolution are stacked on top of each other (i.e., concatenation) and fed into the fully connected layers.

### Model development and ConvGNN hyper-parameters optimization

To ensure model stability, we use 4-fold cross-validation We randomly sample 20% of samples as a testing set. We randomly split the remaining 80% sample into 4 folds, where 3 folds serve as the training set and the remaining one fold serves as the validation set as shown in S1 Fig in S1 File. We cycle through the folds, where each fold serves as a validation set and the remaining 3 folds are used for training. The model parameters were tuned with *optuna* [45] python library [45] based on the model performance on the validation dataset. The hyper-parameter candidate choices were based on Rhee *et al*.’s study and listed in the S1 Table in S1 File. The general grid search algorithm explores the possible combinations of hyper-parameters.

With a grid hyperparameter search with the *optuna*, we chose two convolution layers and 3 fully-connected layers (S2A Fig in S1 File). The number of parameters in each layer is shown in S2B Fig in S1 File. Specifically, the input signal *X* of each omics data is normalized by a batch normalization method [46, 47] to make the learning process stable. Then, the normalized input signal is filtered by a graph convolution layer. Two graph convolution layers were used. Each layer has 32 convolution filters. The filter size of the first layer is 10 and the second layer is 2. The Rectified Linear Unit (ReLu) is used after graph convolution [48]. The pooling size is 2 for both of the layers. Of note, the convoluted signal is normalized through a batch normalization method so that the learning process can be accelerated and have a regularization effect. Then, ReLU activation function [48] and average pooling are applied. The procedure from graph convolution to average pooling is defined as graph convolution layer as in previous studies [12, 15]. After two graph convolution layers, a final feature map is used as an input of a fully connected layer and the dimension of extracted features in each layer is listed in S2B Fig in S1 File. Two fully connected layers are used with 64, 64 hidden nodes for each. Then cross-entropy between prediction and classification label is minimized by the *Adam* algorithm [49]. The learning rate = 0.001 with decay rate = 0.95 and decay step = 10 iterations. The model was trained with 25 epochs. To prevent over-fitting, we add the following regularization strategies in the model training process: 1) Dropout rate = 0.3 [50]; 2) *L*2 regularization = 0.0005 [51, 52]; 3) Early stopping based on the model performance on the validation dataset [53, 54].

### Model evaluation

The model is evaluated by prediction accuracy, and F1 score of CV-trained models on the testing dataset. Accuracy is computed as

$$\begin{array}{c} accuracy=\frac{TP+TN}{TP+TN+FP+FN}, \end{array}$$ (3)

where TP, TN, FP, and FN are the number of true positives, true negatives, false positives, and false negatives, respectively. F1 score is computed as

$$\begin{array}{c} F1=\frac{2*Precision*Recall}{Precision+Recall}=\frac{2*TP}{2*TP+FP+FN}, \end{array}$$ (4)

where Precision $=\frac{TP}{TP+FP}$, and Recall = $\frac{TP}{TP+FN}$.

We compare the performance of spectral-based Convolutional Graph Neural Networks with conventional classification methods including Random Forests (RF), Support Vector Machines (SVM), and eXtreme Gradient Boosting (XGB) as well as a regular deep learning method without graph information called Multi-Layer Perceptron (MLP). To illustrate the strength of multi-omics data integration through Convolutional Graph Neural Network, we compare the model performances with single omics data and multiple omics data. Of note, compared to the Convolutional graph neural networks, we use the exact same set of features (single omics data (1183 mRNAs or 1183 proteins), or multiple omics data) except for the protein-protein interaction networks for other algorithms (RF, SVM, XGB, and MLP). The hyper-parameters in other machine learning methods (RF, SVM, XGB, AND MLP) are also tuned based on the same validation dataset as ConvGNN used. We also compare performance using the known PPI or the COPD-associated PPI on the ConvGNN.

### SHAP analysis on the ConvGNN model of two omics and COPD-associated PPI

To explain how the spectral-based Convolutional Graph Neural Network model(s) classifies COPD, we applied SHAP method to identify important genes/proteins and subnetworks [25]. SHAP is a unified approach to interpreting model predictions. The SHAP value is the average contribution of features that are predicted in different situations and it has three desirable properties for model interpretation: local accuracy, missingness, and consistency. The SHAP method has been demonstrated to have better consistency with human intuition than previous approaches including LIME and DeepLIFT [25]. We focused on the ConvGNN model that integrates two omics data and COPD-associated PPI networks because this model achieves the best performance among the evaluated models. We used the training dataset as a background dataset to generate the perturbed dataset required for training the surrogate models. The testing dataset was used to explain the model output. To interpret the model, sampling data points in the neighborhood of the original data point was performed to build surrogate models [25, 55]. 1200 samples were drawn to build the surrogate model for explaining each prediction. We performed SHAP analysis on an AWS instance with R5.2xlarge 8vCPU and 64G RAM provided by NHLBI BioData Catalyst.

### Gene Ontology enrichment analysis on important features identified by SHAP

To illustrate the effectiveness of the SHAP method and identify the associated pathways and networks of important features in the ConvGNN model, we performed Gene Ontology (GO) enrichment analysis with Fisher’s exact test using the *topGO* R package [56] on the top 30 important genes/proteins identified with SHAP value calculation. The background genes/proteins in GO analysis are the initial 1183 genes/proteins. In other words, the reference list is the list of all the genes/proteins used for model training. Gene Ontology (GO) is a well-known framework for supporting the computational representation of biological systems [57] and has often been used to evaluate the quality of newly constructed or reconstructed protein-protein interaction networks [58, 59]. Specifically, a statistical test is performed to examine whether the number of identified genes belonging to a particular gene set/pathway is higher than that expected by random chance, as determined by comparison to a background gene list [57]. The adjusted P values were calculated with Benjamini-Hochberg Procedure for False Positive Rate (FDR) correction. The significant level was set to FDR < 0.05. In a multi-omics study, enrichment can be performed on a single omic data type, such as transcriptomics and metabolomics enrichment analysis separately or multi-omics together with integration, such as transcriptomics and metabolomics joint pathway analysis [60, 61]. The two omics in our study are transcriptomics (mRNAs) and proteomics (proteins). GO annotations are created by associating a gene’s transcript (mRNA) or gene’s product (protein) with a GO term. For each gene, its mRNA and protein were linked to the same GO annotation. Due to the intricate relationship between mRNA and protein and their joint role in regulating gene expression and protein synthesis, it can be difficult to differentiate their individual functions. Therefore, we performed GO enrichment of 30 important gene features including mRNA and protein, but not analyzed them separately.

### Statistical software

Unless otherwise specified, the data manipulation and data analyses were performed using Python [62]. The Python libraries *Tensorflow*_1.11.0 [63], *optuna*_2.10.0 [45], *sklearn*_0.24.5 [64], *pandas*_1.1.5 [65], *numpy*_1.19.5 [66] and *scipy*_1.5.3 [67] were used for data preparation and ConvGNN model development. *SHAP*_0.28.5 [68] was used for explaining the trained Convolution Graph Neural Network models and identifying important genes/proteins for COPD classification.

## Results

### Clinical characteristics of subjects

The samples in this study covered a range of spirometry profiles including normal (296), COPD with all 4 grades of GOLD airflow limitation severity (GOLD 1: 70; GOLD 2: 152; GOLD 3: 80; GOLD 4: 16) (Table 1). There are differences in age, gender BMI, neutrophil percent, lymphocyte percent, FEV1pp, and Percent emphysema in different groups (p-value < 0.05). However, race is not statistically different (p-value > 0.05).

**Table 1:** Clinical characteristics of overlapping proteomics and transcriptomics dataset.

	GOLD COPD stage					P*	Control	COPD (2-4)	P**
	0	1	2	3	4				
n	296	70	152	80	16		296	248	
gender = Male (%)	132 (44.6)	40 (57.1)	91 (59.9)	45 (56.2)	8 (50.0)	0.021	132 (44.6)	144 (58.1)	0.002
race = White (%)	264 (89.2)	64 (91.4)	145 (95.4)	77 (96.2)	16 (100.0)	0.059	264 (89.2)	238 (96.0)	0.005
Age (mean (SD))	65.34 (8.63)	67.96 (7.66)	71.32 (8.07)	71.11 (7.74)	69.16 (8.64)	<0.001	65.34 (8.63)	71.11 (7.98)	<0.001
BMI (mean (SD))	29.47 (5.89)	27.30 (4.12)	28.47 (5.70)	28.36 (7.30)	28.19 (7.56)	0.055	29.47 (5.89)	28.42 (6.35)	0.046
Current smoker (%)	81 (27.4)	20 (28.6)	30 (19.7)	9 (11.2)	1 (6.2)	0.007	81 (27.4)	40 (16.1)	0.002
Percent of neutrophil (mean (SD))	59.93 (9.01)	61.73 (7.96)	63.25 (8.89)	64.50 (8.41)	68.12 (7.40)	<0.001	59.93 (9.01)	63.97 (8.70)	<0.001
Percent of lymphocyte (mean (SD))	28.47 (8.62)	26.80 (7.66)	24.88 (7.79)	23.41 (7.20)	21.06 (7.24)	<0.001	28.47 (8.62)	24.16 (7.61)	<0.001
Percent of eosinophil (mean (SD))	2.76 (2.09)	2.89 (2.17)	2.87 (1.98)	2.92 (2.13)	2.19 (1.22)	0.713	2.76 (2.09)	2.84 (1.99)	0.625
FEVlpp (mean (SD))	98.25 (13.19)	88.45 (12.90)	61.50 (14.67)	39.39 (11.52)	27.41 (10.52)	<0.001	98.25 (13.19)	52.19 (18.04)	<0.001
Percent of emphysema, (mean (SD))	2.06 (2.72)	7.18 (6.86)	9.68 (9.34)	19.70 (13.94)	29.54 (11.07)	<0.001	2.06 (2.72)	14.20 (12.67)	<0.001

Notes: Data is presented as the mean (standard deviation) for age, body mass index (BMI), FEV1pp percent of neutrophil, percent of lymphocyte, percent of eosinophil, and percent emphysema.

COPD: Chronic Obstructive Pulmonary Disease.

GOLD: the Global Obstructive Lung Disease system for grading COPD severity: GOLD 1 is mild COPD, GOLD 2 is moderate COPD, GOLD 3 is severe COPD, GOLD 4 is very severe COPD, and GOLD 0 is an individual at-risk stage (chronic smoker) but without COPD (control).

FEV1pp: percent predicted forced expiratory volume in one second.

Percent emphysema: percent of lung voxels less than —950 Hounsfield Units on inspiratory CT scans.

P^*^: p values for all GOLD status comparisons.

P^**^: p values for case and control comparisons.

Because there is uncertainty on the COPD diagnosis with GOLD stage 1, we removed those subjects for further analysis in classification model development and evaluation. We categorized the samples into two groups: controls and COPD cases (GOLD = 2—4) (Table 1). There are differences in age, gender, race, smoking status BMI, neutrophil percent, lymphocyte percent, FEV1pp, and Percent emphysema between the two groups (p-value < 0.05) while the percent of eosinophils is not statistically different (p-value > 0.05).

### Graph Convultional Neural Network outperforms conventional methods with single omics data

**Fig 2:**
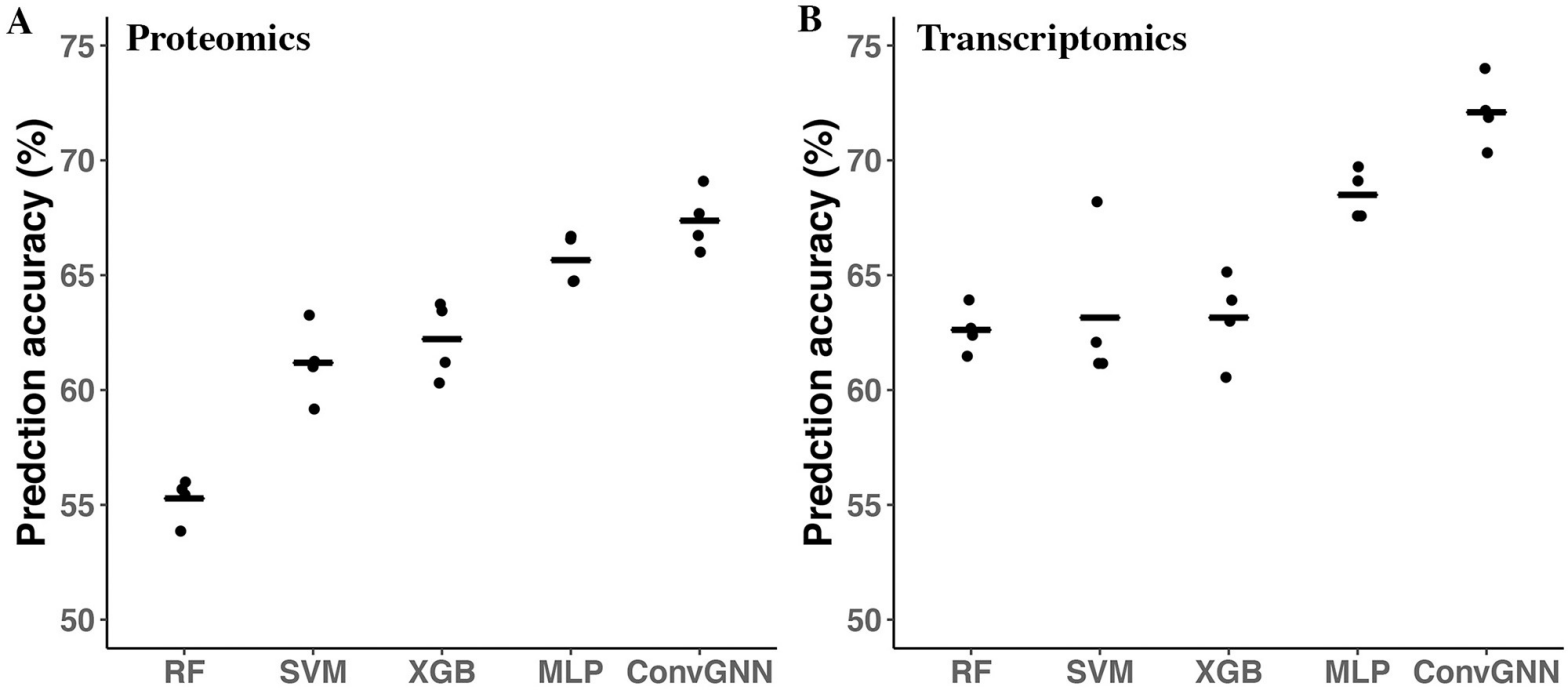
Convolutional Graph Neural Network performance on single omics data. The ConvGNN models were trained in a 4-fold CV strategy with single omics data: proteomics data (A) or transcriptomics data (B). The STRING PPI network was used for the graph convolution. Four other classification methods were also evaluated: RF, SVM, XGB, and MLP. The model performances are assessed using the prediction accuracies on the testing dataset. The lines represent the mean accuracies for CV-trained models and the error bars represent the standard error of the mean.

First, we trained the ConvGNN models on single omics data along with the general PPI network retrieved from the STRING database. A typical learning curve of ConvGNN on the validation dataset with proteomics data was shown in S3 Fig in S1 File. We chose the optimal model based on the model performance on the validation set. For the proteomics data, we found that the ConvGNN model has significantly higher prediction accuracy and F1 score than other tested methods. The average prediction accuracies of the four methods that only used the proteomic data were 55.28 ± 0.94, 61.19 ± 1.69, 62.21 ± 1.70, and 65.66 ± 1.13 for RF, SVM, XGB and MLP respectively. ConvGNN, which incorporated the prior knowledge of PPI and protein expression data achieved higher accuracy of 67.38 ± 1.29 (S2 Table in S1 File), which was statistically significant than all other methods (all P < 0.05, paired student’s t-test) (Fig 2A). The testing dataset is well-balanced and the F1 score results follow a similar pattern to the accuracy (S4 Fig in S1 File).

For the transcriptomics data, we also found that the ConvGNN had significantly higher prediction accuracy than the other testing methods. The average prediction accuracies for RF, SVM, XGB and MLP were 62.62 ± 1.01, 63.15 ± 3.39, 63.15 ± 1.94, and 68.50 ± 1.09, respectively, while ConvGNN with both PPI information and RNAseq expression data achieved 72.09 ± 1.51 accuracy. The differences between ConvGNN and the other 4 methods are statistically significant (all P < 0.05, paired student’s t-test) (Fig 2B).

### Multi-omics integration through ConvGNN increases prediction accuracy

**Fig 3:**
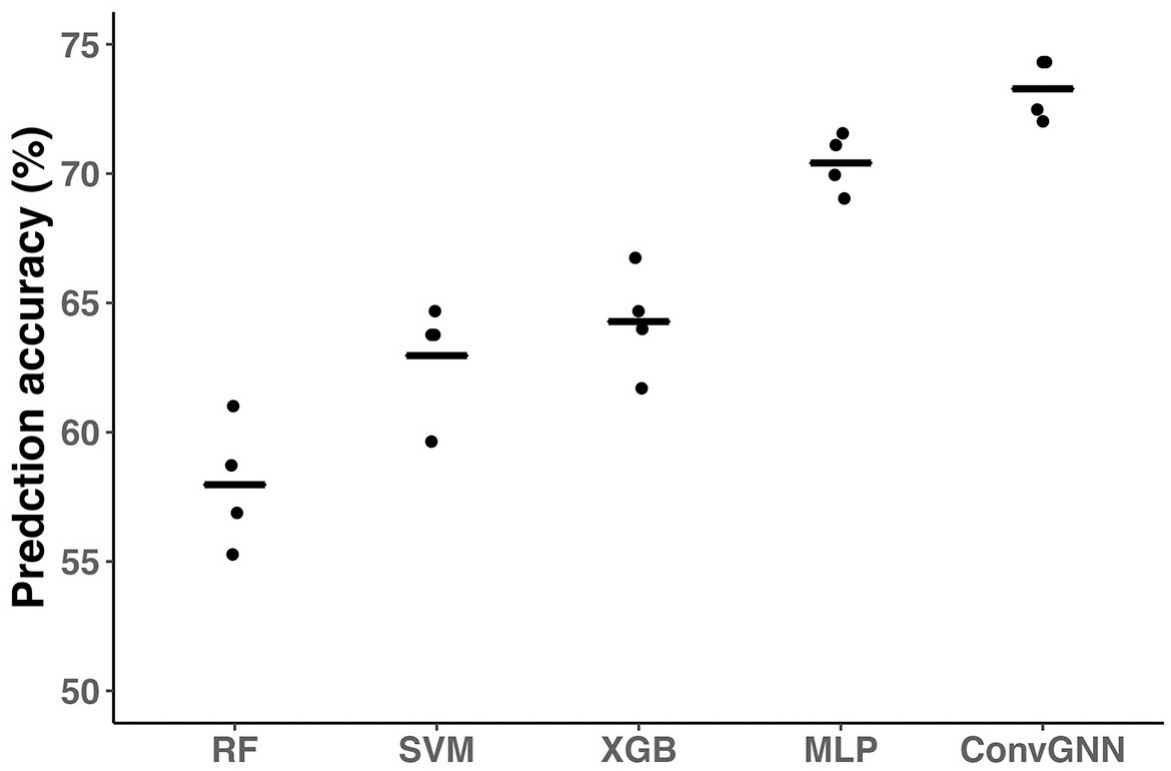
Multi-omics data integration through ConvGNN for COPD prediction. The ConvGNN models were trained in a 4-fold CV strategy with two omics data: proteomics and transcriptomics. The STRING PPI network was used for graph convolution. Besides ConvGNN, we also developed classification models with Random Forest (RF), Support Vector Machine (SVM), eXtreme Gradient Boosting (XGB), and Multi-Layer Perceptron (MLP) for comparison. The model performances are assessed using the prediction accuracies on the testing dataset. The lines represent the mean accuracies for CV-trained models.

**Fig 4:**
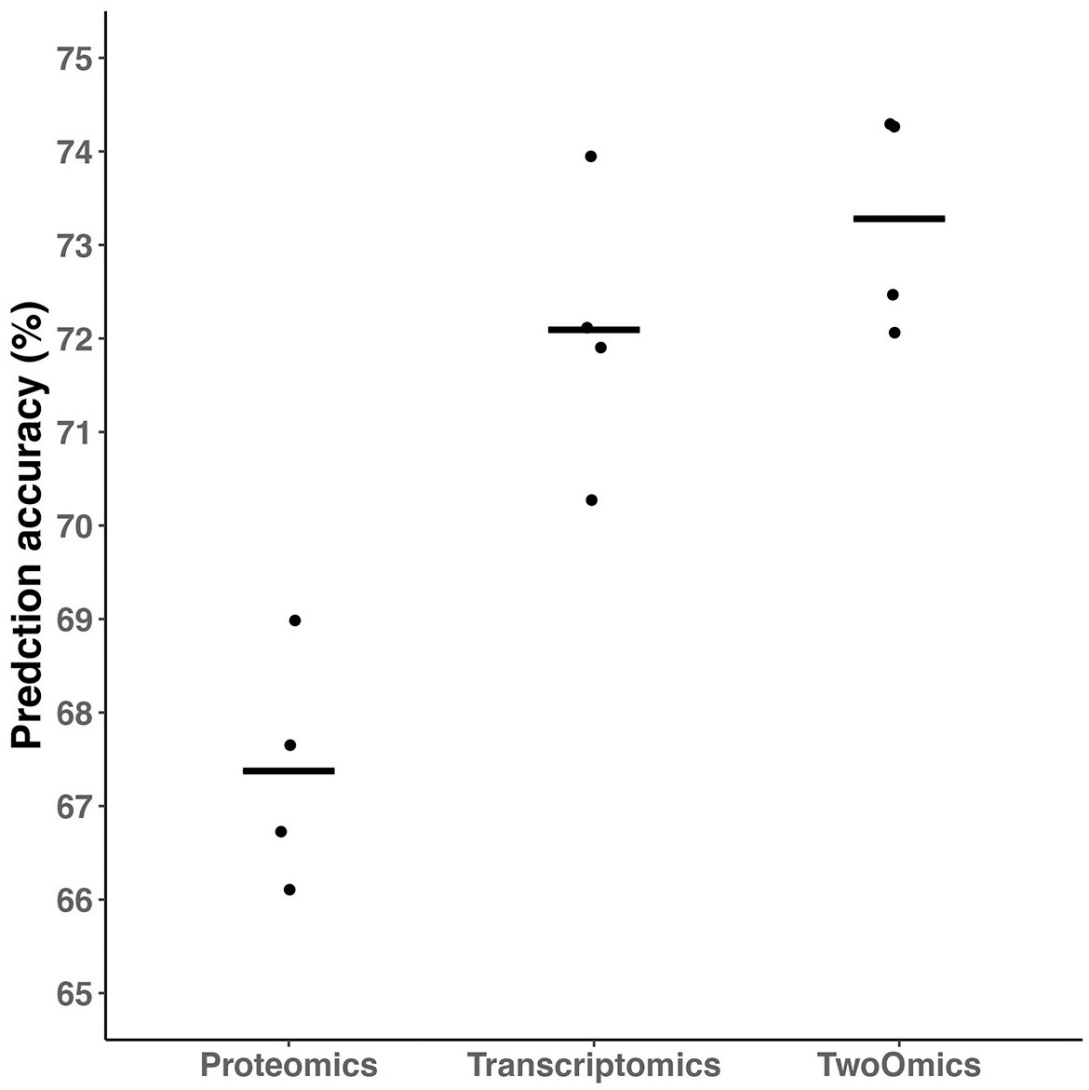
ConvGNN performance with STRING PPI on single omics and multi-omics data. The ConvGNN models were trained in a 4-fold CV strategy as above on the proteomics data (A), transcriptomics data (B), or both (C). The PPI network for ConvGNN was retrieved from the STRING database. The model performances are assessed using the prediction accuracies on the testing dataset. The lines represent the mean accuracies for CV-trained models.

Next, we extended the ConvGNN model to integrate two omics datasets with the general PPI network retrieved from the STRING database. To reduce the computational burden, we had the proteomics and transcriptomics data share the same convolution filters for feature extraction. For fair comparisons with other classification methods, we concatenated the two omics datasets for training RF, SVM, XGB, and MLP models. We found that the ConvGNN model has significantly higher prediction accuracy and F1 score than the other testing methods. The average prediction accuracies for RF, SVM, XGB and MLP were 57.97±2.47, 62.96±2.26, 64.28±2.08, and 70.41±1.14, respectively. The ConvGNN which incorporated the prior knowledge of PPI and proteomic data achieved 73.28±1.20 accuracy (S2 Table in S1 File). The differences between ConvGNN and the other 4 methods are statistically significant (all P < 0.05, paired student’s t-test) (Fig 3). We found that the ConvGNN model with two omics datasets also has significantly higher prediction accuracy than the ConvGNN with a single omics dataset only (all P < 0.05, paired student’s t-test) (Fig 4).

### COPD-associated PPI with AhGlasso improves prediction accuracy

We next investigated the effects of incorporating the COPD-associated PPI network on the ConvGNN. Using the previously known PPI network from the STRING database, we applied AhGlasso on the transcriptomics data of 2656 subjects without proteomics measurements to construct COPD-associated networks. The optimal regularization parameter, λ, was selected to be 0.029 based on BIC. The resultant COPD-associated network has 0.033 density while the density of the original corresponding PPI from STRING is 0.046. The COPD-associated PPI serves as network information for further Graph Neural Network model development.

**Fig 5:**
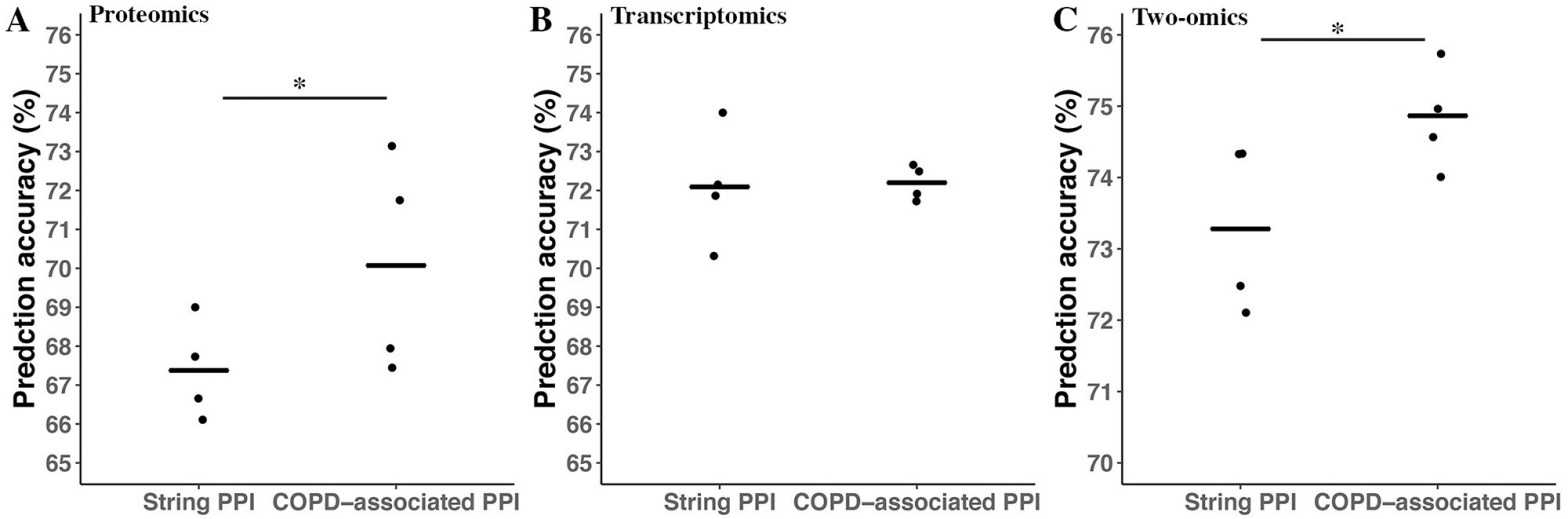
ConvGNN performance with COPD-associated PPI by AhGlasso. The ConvGNN models were trained in a 4-fold CV strategy as above on the proteomics data (A), transcriptomics data (B), or both (C). The PPI network for ConvGNN was either retrieved from the STRING database or COPD-associated PPI with AhGlasso. The model performances are assessed using the prediction accuracies on the testing dataset. The lines represent the mean accuracies for CV-trained models. The differences between conventional classification models are tested with paired student’s t-test (*, P ≤ 0.05).

With proteomics data and COPD-associated PPI, the average prediction accuracy of ConvGNN achieves 70.07 ± 2.84 while the average prediction accuracy of ConvGNN with the STRING PPI is 67.38 ± 1.29 (P = 0.05, paired student’s t-test) (Fig 5A). With transcriptomics data, the average prediction accuracy of ConvGNN achieve to 72.20 ± 0.44 while the average prediction accuracy of ConvGNN with the STRING PPI is 72.09 ± 1.50 (P = 0.91, paired student’s t-test) (Fig 5B). With proteomics data, transcriptomics data, and COPD-associated PPI, the average prediction accuracy of ConvGNN is 74.86 ± 0.67 while the average prediction accuracy of ConvGNN with the STRING PPI is 73.28 ± 1.20 (P = 0.045, paired student’s t-test) (Fig 5C, S2 Table in S1 File). The prediction results for each GOLD level subject in the ConvGNN model with two omics and COPD-associated are presented in S3 Table in S1 File. Of note, the prediction accuracy for GOLD 2 subjects is 65.71% while the accuracy for GOLD 3 and 4 is 90% and 100%, respectively.

### Identifying most important features through SHAP

**Fig 6:**
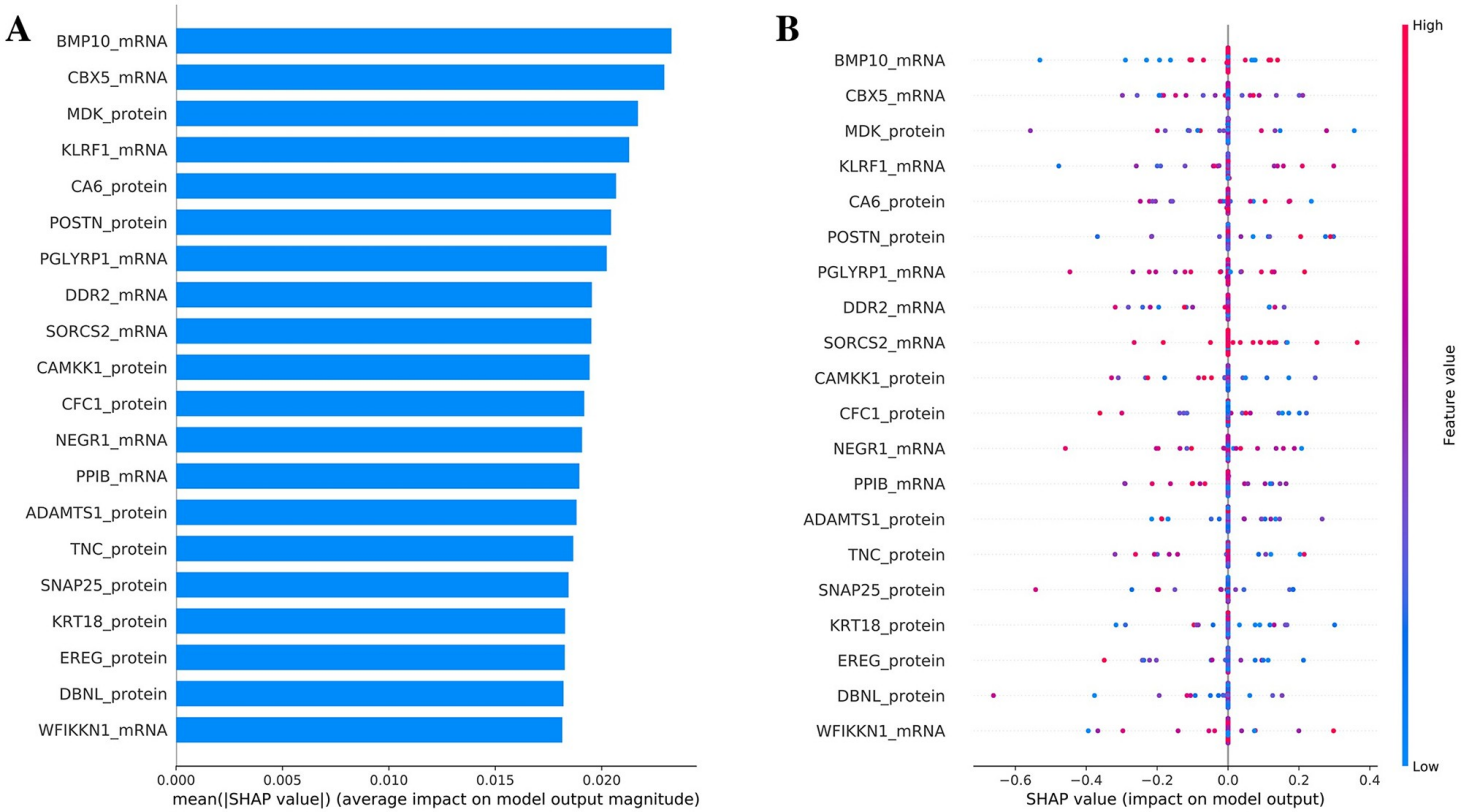
Top 20 important features identified with SHAP values. The SHAP values were calculated on the testing dataset with 1200 samplings. The feature importance is evaluated based on the average absolute SHAP values over subjects. The top important features are ranked in descending order. (A) The horizontal bars show the average impact of a feature on model output magnitude. (B) Impact of top 20 important features on the model output. Each dot represents each subject. The dot color shows whether that feature (variable) is high (in red) or low (in blue) for that observation. The horizontal location shows whether the effect of that value is associated with a higher or lower prediction.

To better explain how the spectral-based convolutional Graph Neural Network model(s) works, we perturbed features and estimate the marginal contributions of different features through the SHAP analysis. We focused on the ConvGNN model that integrates two-omics data and COPD-associated PPI networks reconstructed by the AhGlasso algorithm because this model achieved the highest accuracy among the models evaluated. Based on the mean absolute value of the SHAP values for each feature across all samples in the testing dataset, we identified the top important genes/proteins for COPD prediction in the ConvGNN model based on the marginal contribution of each feature, which helps interpret the model globally. With the SHAP library default setting, we further explore the top 20 features (Fig 6A and 6B) and examine the impact of each feature on the model output. The top 20 important genes and proteins for the ConvGNN model are a mix of genes and proteins, which suggests that there is no single omics dominating in the model for prediction. Also, there are no overlapping genes and proteins on the top 20 important features. In addition, we also apply the SHAP method to identify the important features in the other 4 methods (RF, SVM, XGB, and MLP). There are only a few overlapping top 30 important features in the 5 predictive models (S5 Fig in S1 File), which is partially due to mathematical differences among different approaches.

**Fig 7:**
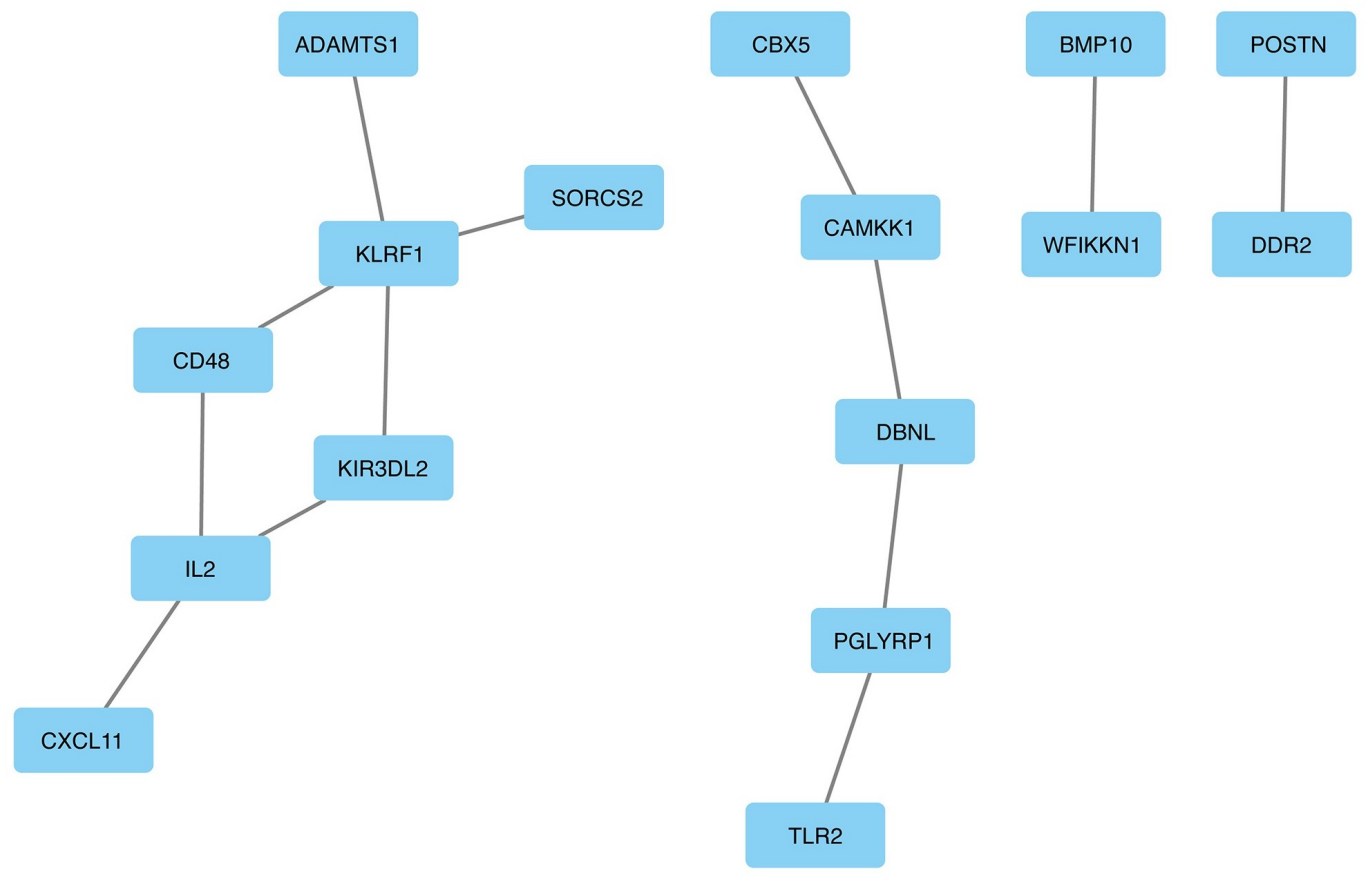
Important subnetworks for COPD prediction. Top important genes/proteins are identified with SHAP values. The sub-adjacency matrix of the top 30 important genes/proteins is extracted for plotting. The genes/proteins without any connections are removed.

The protein-protein interactions of the top 30 genes/proteins (S4 Table in S1 File) were extracted from the COPD-associated PPI network and presented in Fig 7. Of note, we chose the top 30 important genes and proteins instead of 20 to extract robust subnetworks. There are 4 important subnetworks for COPD prediction. One subnet consists of CXCL11, IL-2, CD48, KIR3DL2, KRF1, ADAMTS1, and SORCS. Another subset includes TLR2, PGLYRP1, DBNL, CAMKK1, and CBX5. The other subnetworks include two genes/proteins: 1) BMP10 and WFIKKN1; 2) POSTN and DDR2.

**Fig 8:**
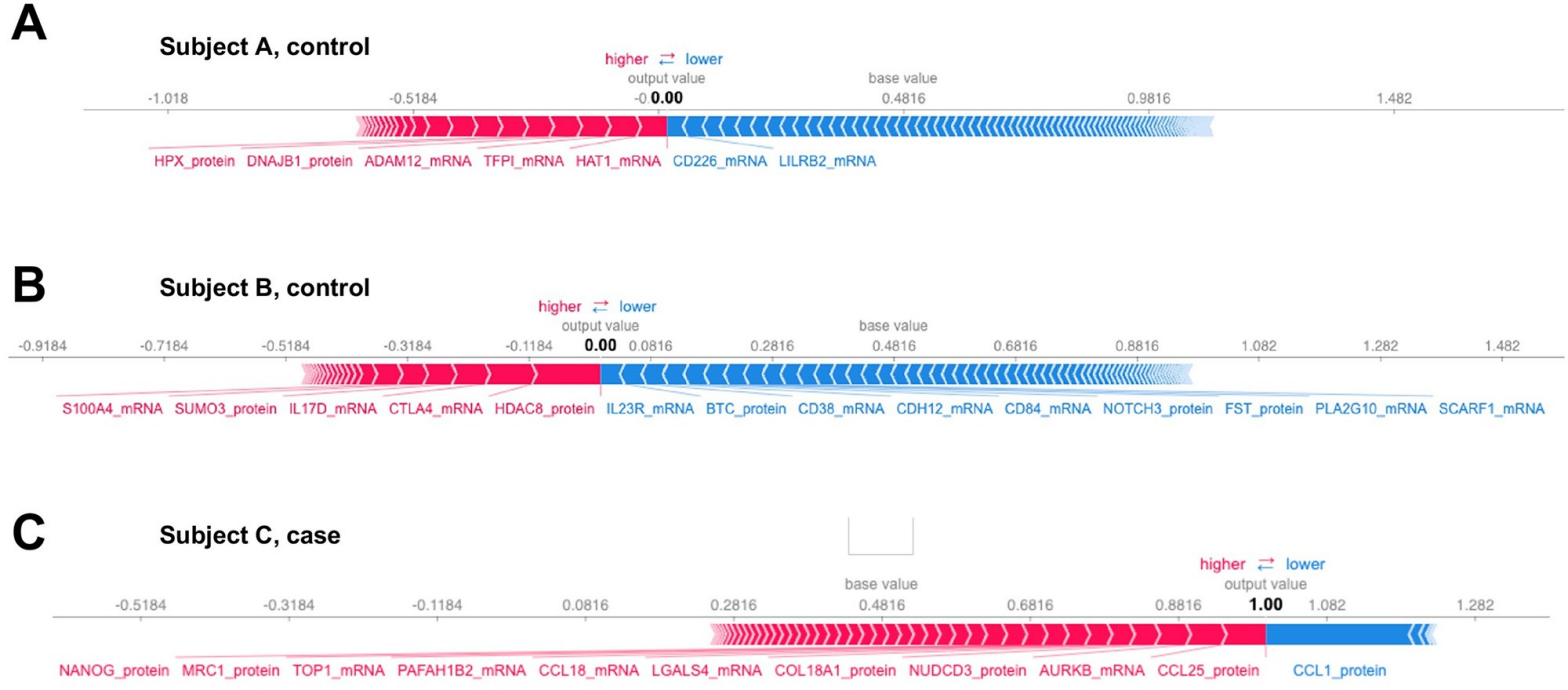
Important features on an individual subject. The SHAP values were calculated on two subjects with 1200 samplings to illustrate the local interpretability: Subject A (A), subject B (B), and subject C (C). Subject A and subject B are healthy controls while subject C is a COPD case. The output value is the prediction for that observation. The base value is the value that would be predicted if we have no feature information (expected value). Features pushing the prediction higher (to the right) are shown in red while those pushing the prediction lower are in blue. The bar length of each feature represents its relative contribution to the final output: a wider bar denotes a larger contribution.

Besides global interpretability, the SHAP method also provides local interpretability for each subject [25]. Specifically, each subject gets its own set of SHAP values. We illustrated the important features and their contribution in terms of SHAP on subjects A, B, and C (Fig 8). For example, in a control subject A, HAT1, TFPI, and ADAM12 transcripts push the prediction value higher while CD226 and LILRB2 transcripts push the prediction value lower. The prediction (output value) of subject A in the ConvGNN model is 0, which represents “healthy control”. In COPD subject C, CCL25, ARRKB, LGALS4, and other genes/proteins push the prediction value higher while CCL1 pushes the prediction value lower. The prediction of subject C in the ConvGNN model is 1, which represents “COPD”.

### GO enrichment analysis on top important genes

We analyzed the enrichment gene ontology (GO) terms and biological pathways of the top 30 important features including genes and proteins (S4 Table in S1 File), which were discovered through SHAP analysis. We chose the top 30 important genes and proteins instead of 20 for GO enrichment analysis to ensure the enrichment findings are more robust. Of note, there are no overlapping genes and proteins on the top 30 important features. For example, BMP10_mRNA was in the top 30 important features, but not BMP10_protein. We found that 6 enriched molecular function pathways (Table 2), including glycosaminoglycan binding signaling and heparin signaling pathways, which have been reported to be important for COPD pathogenesis [69, 70]. We also found 47 enriched biological process and 16 enriched cellular component pathways (S5 and S6 Tables in S1 File).

**Table 2:** GO enrichment of the top 30 important genes/proteins in the COPD ConvGNN model.

GO.ID		Term	Annotated	Significant	Expected	P
1	GO:0005539	glycosaminoglycan binding	98	8	2.5	0.0022
2	GO:0008201	heparin binding	74	6	1.89	0.0089
3	GO:0097367	carbohydrate derivative binding	290	13	7.39	0.0179
4	GO:1901681	sulfur compound binding	86	6	2.19	0.0182
5	GO:0033612	receptor serine/threonine kinase binding	10	2	0.25	0.0249
6	GO:0033218	amide binding	47	4	1.2	0.0285

Notes:

Annotated, number of proteins in a pathway from the complete set of 1183 proteins;

Significant, number of proteins in a pathway from 30 top important genes/proteins;

Expected, the expected number of proteins in a pathway if we randomly selected 30 proteins from 1183 background proteins;

P: P value of Fisher’s exact test

## Discussion

In this study, we first successfully developed the ConvGNN models to incorporate known protein-protein interaction networks with single omics data (proteomics or transcriptomics). Similar to [15], the ConvGNN models outperform many existing methods including RF, SVM, XGB, and MLP. This finding demonstrates the advantages of incorporating PPI information. In Rhee *et al*.’s study, a hybrid model including graph convolution neural network (ConvGNN) and relation network (RN) was also proposed [15]. Although we tried this hybrid method, we did not find a significant improvement in terms of prediction accuracy after adding the relation network component, which might be partially explained by the increase of parameters to be trained in a more complex model, which often requires a large dataset and can be challenging to train.

Next, we extended the ConvGNN approach for multi-omics data integration. For model simplicity and to reduce computation burden, we assume the interaction relationships among transcripts and proteins are similar and the convolution filters could be shared. We demonstrate that the extended ConvGNN successfully incorporates two omics data and PPI knowledge and improves prediction accuracy in COPD, which suggests that the ConvGNN could be a good approach for multi-omics data and network integration.

Although the ConvGNN method with the PPI from the STRING database outperforms many existing methods, the STRING PPI is general but not specific to COPD. We reconstructed the COPD-associated PPI network through the AhGlasso algorithm based on one independent transcriptomics dataset including COPD cases and controls. We found that this newly-COPD-associated PPI improves the prediction performance of ConvGNN models trained either with the proteomics dataset only or two omics datasets together. However, we did not observe the improvement of ConvGNN based on transcriptomics data. The lack of improvement in this case may be because the COPD-associated PPI was reconstructed based on the held out transcriptomics data, which might contain similar information to the included transcriptomics data, even though the samples are independent of each other.

For downstream analysis, SHAP provides a unified framework to interpret machine learning models and measures a feature’s importance by calculating the increase of the model’s prediction error after perturbing the feature. The SHAP analysis has provided two great advantages: 1) Global interpretability: the SHAP values can show how much each gene/protein contributes to the ConvGNN model; 2) Local interpretability: each subject in the testing dataset gets its own set of SHAP values [25]. In addition, the SHAP analysis also identifies the top important genes/proteins that have a large impact on the ConvGNN prediction of COPD. Among the top 30 gene/proteins, we found significantly enriched glycosaminoglycan, heparin signaling, and carbohydrate derivative signaling pathways. These three pathways have been demonstrated to play important roles in COPD [71–75]. We also found 4 important subnetworks for COPD prediction. One subnet consists of CXCL11, IL-2, CD48, and KIR3DL2. They have been demonstrated to interact together and play important role in the regulation of the inflammatory and immune responses [76, 77], which play key roles in the development and progression of COPD [78]. Another subset includes TLR2, PGLYRP1, DBNL, CAMKK1, and CBX5. It has been demonstrated that TLR2 (Toll-like receptor 2) plays an important role in the immunoregulation of the inflammatory process in COPD and is involved in the development of COPD exacerbation [79]. The other subnetworks include two genes/proteins: 1) BMP10 and WFIKKN1; 2) POSTN and DDR2. BMP10 is currently viewed as one of the major molecules playing a critical role in pulmonary hypertension, which is a common complication of COPD [80, 81]. Besides the SHAP approach, LIME also can provide local explanations for a particular subject. We found there are only a few overlapping top features identified with two explainers: SAHP and LIME. The difference might be due to the measurement difference for feature importance: LIME fits a simple model around a prediction to create a local explanation while SHAP uses game theory to measure the importance of each feature.

Despite the advantages of incorporating the COPD-associated PPI and omics data in the ConvGNN for predicting COPD, there are some limitations to this study. One limitation is that the prediction accuracy is not very high, which might be partially caused by the small sample size. ConvGNN training involves a large number of parameter optimizations, which is best suited for larger sample sizes. In this study, we extend the ConvGNN to integrate multi-omics data and only focus on the subjects with both proteomics and transcriptomics measurements in the COPDGene Phase II study, which limits the amount of available data. Although the data augmentation technique has been used to increase the size of data used for training a model for image classification and natural language processing [82, 83], it is still not clear how to augment the omics data and disease outcome simultaneously because PPI networks are vast and complicated representations of biological processes. Another limitation is the age difference between the control and COPD groups. We only included omics data and not demographic variables in the predictive model. In the future, as larger omic data sets in COPD are available, we will be able to assess generalizability in other cohorts that may have more variability in ages between groups. In addition, how changes in transcript/proteins profiles affect complex heterogeneous diseases through these interactions is still not clear [84]. Another limitation is that we only have 1183 overlapping transcripts/proteins for model development. The limiting factor is that the SomaScan® 1.3k assay only detects 1.3k proteins and protein complexes. SomaScan® recently released a new assay (7k) that could detect up to 7000 proteins and protein complexes, which would increase the overlap with the RNAseq data and allow for a larger network for the ConvGNN. In addition, we used all overlapping features between the two omics data, which might include irrelevant features and noise signals to inhibit model performance. Similarly, the new 7k platform may also introduce additional noisy signals. In the future, we plan to select COPD-related genes/proteins based on extensive literature search and review and use them for ConvGNN model(s) as Rhee *et al*.’s study, where they only focused on a set of known cancer-related genes. Finally, another limitation is that the convolution kernels for the two omics data were set to be the same to reduce the computational burden. However, the interactions between mRNA transcripts and the interactions between proteins might be different and require differenet kernels.

Regarding interpretation, although SHAP provides several desirable properties and advantages to explain regular machine learning models, it ignores the interaction relationship of genes and proteins during feature perturbation, which could limit its capacity to interpret the ConvGNN accurately. In the future, if the SHAP value could be evaluated with the guidance from the PPI network, we may have improved power to identify important genes/proteins and discover relevant biological pathways in the ConvGNN model for disease classification. In addition, it is an approximation based on feature perturbation and sampling, which is computation-intensive and memory demanding. Although we tried to increase the sampling number to better estimate the feature contributions, we faced memory issues. Therefore, the estimation of feature importance in our study might be biased.

## Conclusion

In this study, we integrated single omics data (proteomics data and transcriptomics data individually) with a general PPI network from the STRING database and successfully developed ConvGNN models for COPD classification to outperform several conventional classification methods. Then we reconstructed the COPD-associated PPI network through the AhGlasso algorithm based on an independent transcriptomics dataset including COPD cases and controls. With the COPD-associated PPI network, we further extended the ConvGNN method for incorporating transcriptomics and proteomics data and the ConvGNN with two omics datasets improves prediction accuracy over the model with single omics data only. The updated COPD-associated network with AhGlasso further improves the model performance. Although we focused on the spectral-based GNN model development for COPD, the spatial-based Graph Neural Network and/or Graph Attention model might also have great potential in predicting COPD and identifying key subnetworks associated with COPD.

In many cases, interpretability is an important quality for machine learning models used for disease diagnosis. In this study, we explained how the ConvGNN model works globally with the marginal contribution of each feature (global interpretability) and how each observation derives its own prediction by providing an individual set of SHAP values (local interpretability). We have identified CXCL11, IL-2, CD48, KIR3DL2, TLR2, BMP10 and several other relevant COPD genes in subnetworks of the ConvGNN model for COPD prediction. Finally, Gene Ontology (GO) enrichment analysis identified glycosaminoglycan, heparin signaling, and carbohydrate derivative signaling pathways significant enriched in the top important gene/proteins for COPD classifications.

## Supporting information

S1 File (PDF)

S1 Table (TEX)

S2 Table (TEX)
