# Supplementary material for: Deep learning on graphs for multi-omics classification of COPD
Source: PLoS One. 2023 Apr 21;18(4):e0284563. doi: 10.1371/journal.pone.0284563 (PMC10121008; doi:10.1371/journal.pone.0284563)
Supplement: S1 File — (PDF) [file pone.0284563.s001.pdf]

1 SUPPLEMENTARY TABLES

Table S1. Summary of hyper-parameter candidates for grid search

Hyper parameters	Candidates
**Convolution layers (CN):**	
CN: number of convolution	1, 2, 3
F: features	16, 32, 64
K: filter sizes	2, 6, 10
Pooling	max, average
Pooling size	2
**Full-connected layers (MLP):**	
FN: number of fully connected layers	2, 3, 4
M: number of hidden neurons	32, 64, 128, 256
**Epoch**	10, 25, 40, 60, 100
**Learning rate**	0.001, 0.003, 0.005
**Decay rate**	1, 0.95, 0.9, 0.8
**Decay steps**	10, 20
**Momentum**	Adam
**Regularization**	0.0005, 0.001
L2	0.005, 0.001
Droppout	0.1, 0.2, 0.3

Table S2. Summary of prediction accuracy of training models on the testing dataset in different scenarios.

method	proteomics data	transcriptomics data	two omics data
RF	55.28 ± 0.94	62.62 ± 1.01	57.97 ± 2.47
SVM	61.19 ± 1.69	63.15 ± 3.39	62.96 ± 2.26
XGB	62.21 ± 1.70	63.15 ± 1.94	64.28 ± 2.08
MLP	65.66 ± 1.13	68.50 ± 1.09	70.41 ± 1.14
ConvGNN + string PPI	67.38 ± 1.29	72.09 ± 1.51	73.28 ± 1.20
ConvGNN + COPD-associated PPI	70.07 ± 2.84	72.20 ± 0.44	74.86 ± 0.67

Notes: Data is presented as the mean ± standard deviation of prediction accuracy.

Table S3. Confusion matrix of the ConvGNN with two omics data and COPD-associated PPI

	**Prediction class**		
**Actual Gold status**	**Healthy**	**COPD**	**accuracy (%)**
**0**	50	10	83.33
**2**	12	23	65.71
**3**	1	9	90.00
**4**	0	4	100.00

Table S4. Top 30 features in ConvGNN with two-omics data

	**top 30 featurees in ConvGNN**
1	BMP10_mRNA
2	CBX5_mRNA
3	MDK_protein
4	KLRF1_mRNA
5	CA6_protein
6	POSTN_protein
7	PGLYRP1_mRNA
8	DDR2_mRNA
9	SORCS2_mRNA
10	CAMKK1_protein
11	CFC1_protein
12	NEGR1_mRNA
13	PPIB_mRNA
14	ADAMTS1_protein
15	TNC_protein
16	SNAP25_protein
17	KRT18_protein
18	EREG_protein
19	DBNL_protein
20	WFIKKN1_mRNA
21	EPHA10_protein
22	RTN4R_protein
23	CXCL11_mRNA
24	ANG_mRNA
25	TLR2_protein
26	ARSB_mRNA
27	CD48_mRNA
28	IL11_mRNA
29	KIR3DL2_protein
30	IL2_protein

Table S5. Biological process GO enrichment of the top 30 important genes/proteins in the COPD ConvGNN model

	GO.ID	Term	Annotated	Significant	Expected	P
1	GO:0010975	regulation of neuron projection development	82	7	2.09	0.0034
2	GO:0007292	female gamete generation	26	4	0.66	0.0035
3	GO:0048477	oogenesis	13	3	0.33	0.0036
4	GO:0031344	regulation of cell projection organization	97	7	2.48	0.0088
5	GO:0120035	regulation of plasma membrane bounded cell projection organization	97	7	2.48	0.0088
6	GO:0048468	cell development	300	14	7.66	0.0091
7	GO:0010976	positive regulation of neuron projection development	55	5	1.4	0.0109
8	GO:0045620	negative regulation of lymphocyte differentiation	20	3	0.51	0.0128
9	GO:0045664	regulation of neuron differentiation	104	7	2.66	0.0128
10	GO:0031175	neuron projection development	158	9	4.03	0.0132
11	GO:0007281	germ cell development	22	3	0.56	0.0167
12	GO:0050767	regulation of neurogenesis	136	8	3.47	0.0167
13	GO:0031346	positive regulation of cell projection organization	62	5	1.58	0.0178
14	GO:0060284	regulation of cell development	167	9	4.26	0.0187
15	GO:0048666	neuron development	168	9	4.29	0.0194
16	GO:0010720	positive regulation of cell development	113	7	2.89	0.0198
17	GO:0050769	positive regulation of neurogenesis	90	6	2.3	0.0226
18	GO:0045666	positive regulation of neuron differentiation	66	5	1.69	0.0229
19	GO:0042698	ovulation cycle	25	3	0.64	0.0237
20	GO:0060998	regulation of dendritic spine development	10	2	0.26	0.025
21	GO:1900006	positive regulation of dendrite development	10	2	0.26	0.025
22	GO:0120036	plasma membrane bounded cell projection organization	177	9	4.52	0.0268
23	GO:0048589	developmental growth	120	7	3.06	0.0269
24	GO:0030030	cell projection organization	179	9	4.57	0.0286
25	GO:0051606	detection of stimulus	27	3	0.69	0.0291
26	GO:0045687	positive regulation of glial cell differentiation	11	2	0.28	0.03
27	GO:0061036	positive regulation of cartilage development	11	2	0.28	0.03
28	GO:0034103	regulation of tissue remodeling	28	3	0.71	0.032
29	GO:0061844	antimicrobial humoral immune response mediated by antimicrobial peptide	28	3	0.71	0.032
30	GO:0008585	female gonad development	29	3	0.74	0.0351
31	GO:0046545	development of primary female sexual characteristics	29	3	0.74	0.0351
32	GO:1902106	negative regulation of leukocyte differentiation	29	3	0.74	0.0351
33	GO:0009593	detection of chemical stimulus	12	2	0.31	0.0355
34	GO:0030728	ovulation	12	2	0.31	0.0355
35	GO:0045066	regulatory T cell differentiation	12	2	0.31	0.0355
36	GO:0045589	regulation of regulatory T cell differentiation	12	2	0.31	0.0355
37	GO:0051960	regulation of nervous system development	156	8	3.98	0.0359
38	GO:0048699	generation of neurons	219	10	5.59	0.0381
39	GO:0022412	cellular process involved in reproduction in multicellular organism	30	3	0.77	0.0383
40	GO:0060996	dendritic spine development	13	2	0.33	0.0413
41	GO:0051962	positive regulation of nervous system development	104	6	2.66	0.0427
42	GO:0046660	female sex differentiation	32	3	0.82	0.0452
43	GO:0050830	defense response to Gram-positive bacterium	32	3	0.82	0.0452
44	GO:0043616	keratinocyte proliferation	14	2	0.36	0.0474
45	GO:0007276	gamete generation	55	4	1.4	0.0476
46	GO:0030182	neuron differentiation	196	9	5	0.0485
47	GO:1903707	negative regulation of hemopoiesis	33	3	0.84	0.0489

Table S6. Cellular component GO enrichment of the top 30 important genes/proteins in the COPD ConvGNN model

	GO.ID	Term	Annotated	Significant	Expected	P
1	GO:0030427	site of polarized growth	21	4	0.53	0.0015
2	GO:0043204	perikaryon	15	3	0.38	0.0055
3	GO:0044297	cell body	73	6	1.86	0.0082
4	GO:0030426	growth cone	20	3	0.51	0.0126
5	GO:0005604	basement membrane	21	3	0.53	0.0145
6	GO:0043005	neuron projection	117	7	2.97	0.0231
7	GO:0150034	distal axon	25	3	0.64	0.0234
8	GO:0043025	neuronal cell body	67	5	1.7	0.0239
9	GO:0031225	anchored component of membrane	45	4	1.14	0.0245
10	GO:0036477	somatodendritic compartment	92	6	2.34	0.0245
11	GO:0042995	cell projection	181	9	4.6	0.0298
12	GO:0030425	dendrite	50	4	1.27	0.0346
13	GO:0097447	dendritic tree	50	4	1.27	0.0346
14	GO:0097060	synaptic membrane	29	3	0.74	0.0347
15	GO:0005802	trans-Golgi network	13	2	0.33	0.041
16	GO:0045335	phagocytic vesicle	13	2	0.33	0.041

2 SUPPLEMENTARY FIGURES

Figure S1. Flowchart of classification models development and assessment

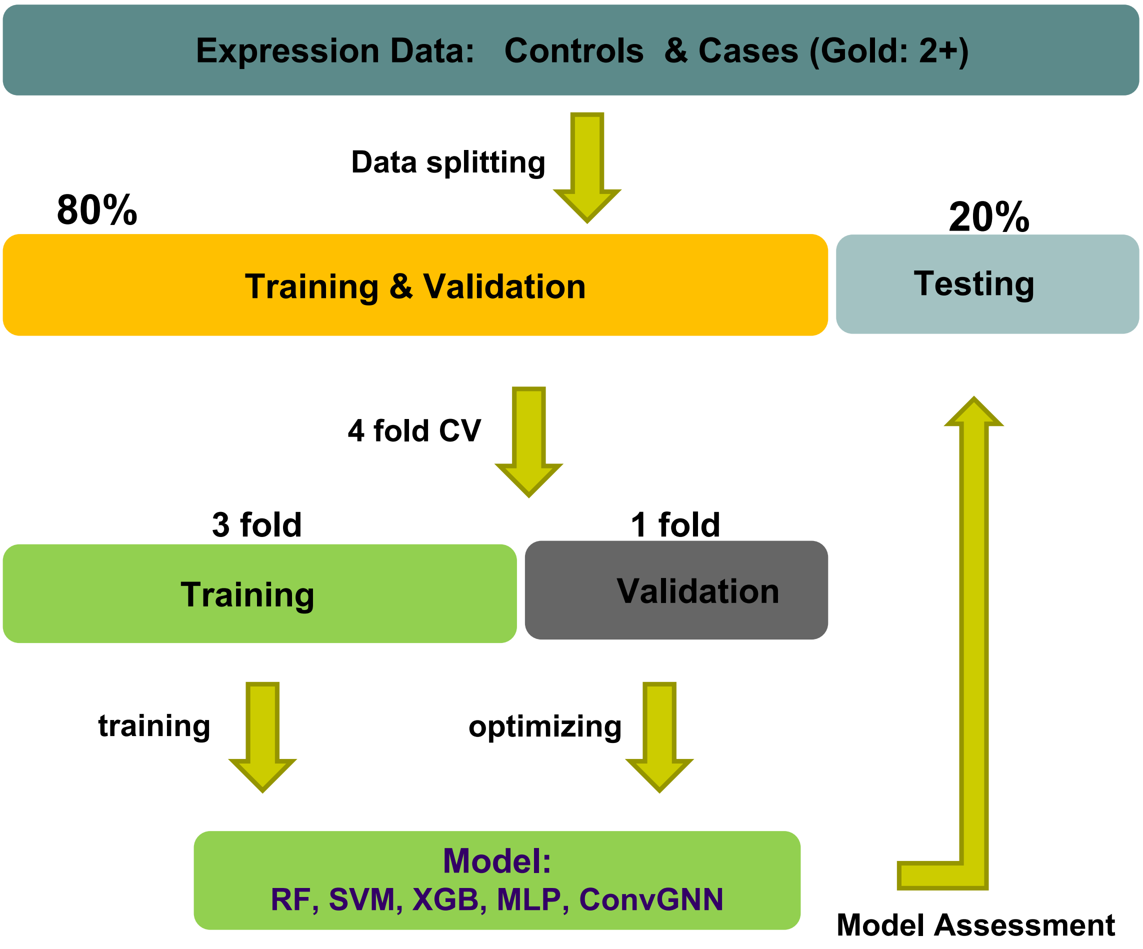

Figure S1: Flowchart of classification models development and assessment. We randomly sample 20% of samples as a testing set. Using 4-fold cross-validation (CV), we randomly split the remaining 80% sample into 4 folds. In each iteration, 3 folds serve as the training set and the remaining one fold is used for validation and parameter tuning. The ConvGNN prediction model was developed based on the training dataset and the early stopping model training was based on the prediction accuracy of the validation dataset. Besides ConvGNN, we also developed classification models with Random Forest (RF), Support Vector Machine (SVM), Extreme Gradient Boosting (XGB), and multi-layer perceptron (MLP) for comparison. The model performances are assessed using the averaged accuracies and F1 scores on the testing dataset.

Figure S2. Illustration of spectral-based Convolutional Graph Neural Network architecture.

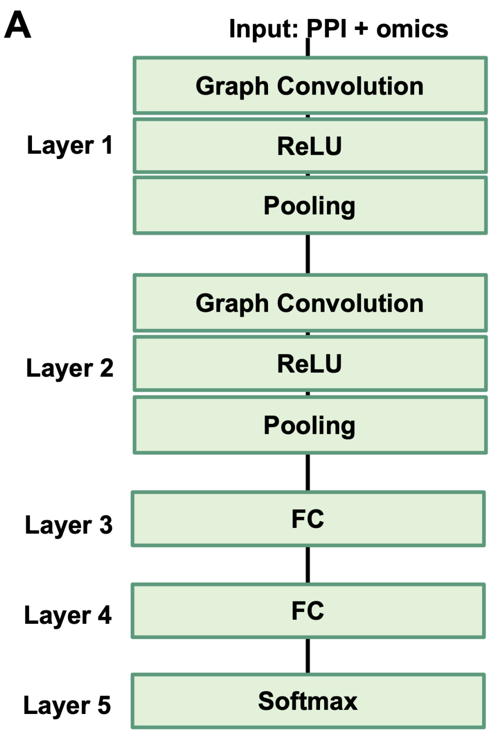

B

```
NN architecture
  input: M_0 = 1208
  layer 1: cgconv1
        representation: M_0 * F_1 / p_1 = 1208 * 32 / 2 = 19328
        weights: F_0 * F_1 * K_1 = 1 * 32 * 10 = 320
        biases: F_1 = 32
  layer 2: cgconv2
        representation: M_1 * F_2 / p_2 = 604 * 32 / 2 = 9664
        weights: F_1 * F_2 * K_2 = 32 * 32 * 2 = 2048
        biases: F_2 = 32
  layer 3: fc1
        representation: M_3 = 64
        weights: M_2 * M_3 = 9664 * 64 = 618496
        biases: M_3 = 64
  layer 4: fc2
        representation: M_4 = 64
        weights: M_3 * M_4 = 64 * 64 = 4096
        biases: M_4 = 64
  layer 5: logits (softmax)
        representation: M_5 = 2
        weights: M_4 * M_5 = 64 * 2 = 128
        biases: M_5 = 2
```

Figure S2: Illustration of spectral-based Convolutional Graph Neural Network architecture. (A) The optimized ConvGNN model for single omics data includes 2 graph-convolution layers, 2 fully-connected (FC) layers, and the last softmax layer. Relu activation denotes rectified linear unit. Softmax activation turns numbers aka logits into probabilities. The right part of the figure details the exact number of parameters in each layer (B).Of note, besides 1183 feature nodes, 25 pseudo nodes with neutral value (0) were added to input matrix (1208 nodes in total) in graph neural network model training to ensure fast pooling of graph signals.

Figure S3. A learning curve of ConvGNN on the validation dataset with proteomics data.

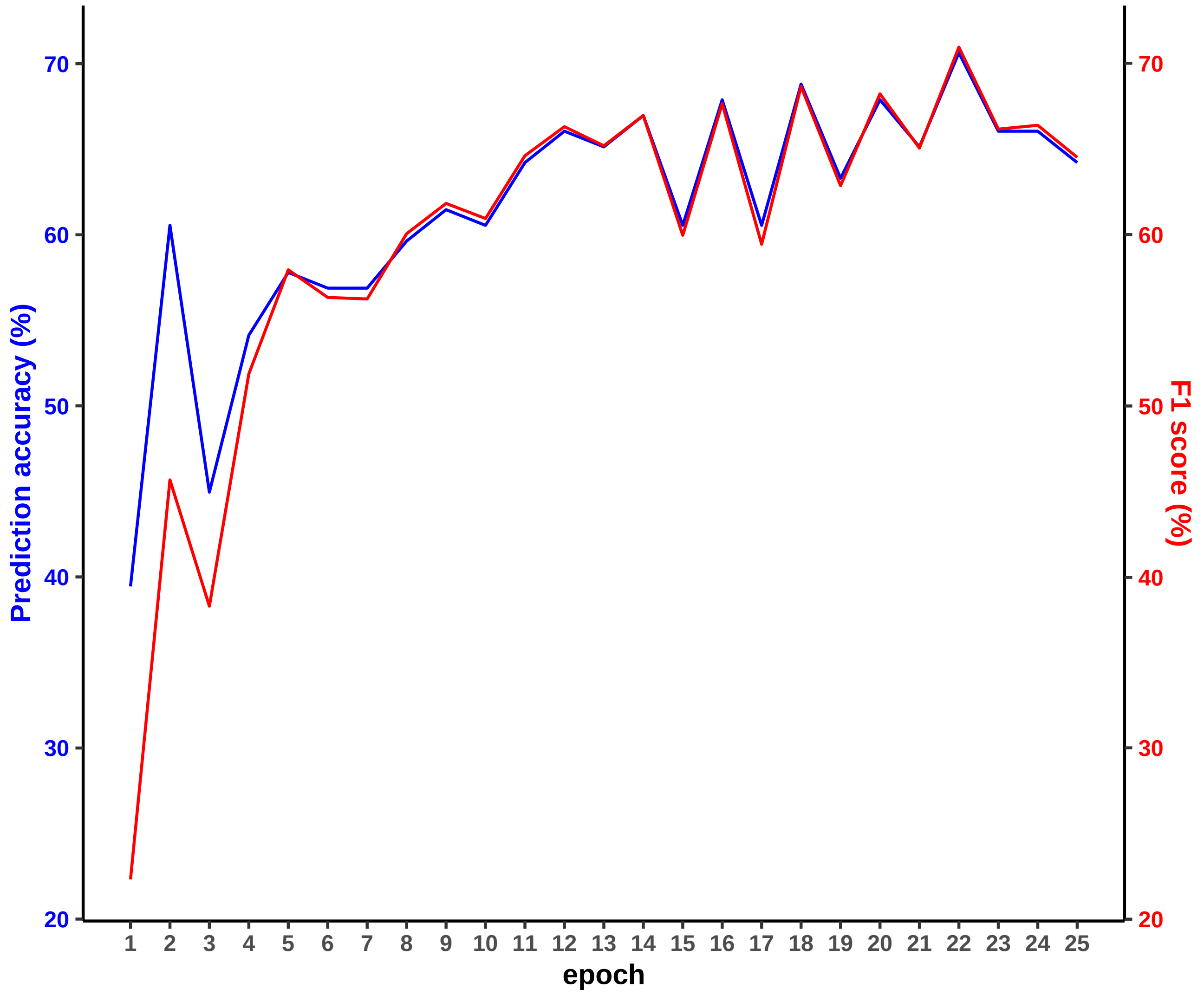

Figure S3: A learning curve of ConvGNN on the validation dataset with proteomics data. The ConvGNN models were trained with proteomics training data with STRING PPI network. The prediction accuracy and F1 score of ConvGNN on the validation datasets were plotted with epochs.

Figure S4. F1 score comparison between ConvGNN and the other 4 approaches.

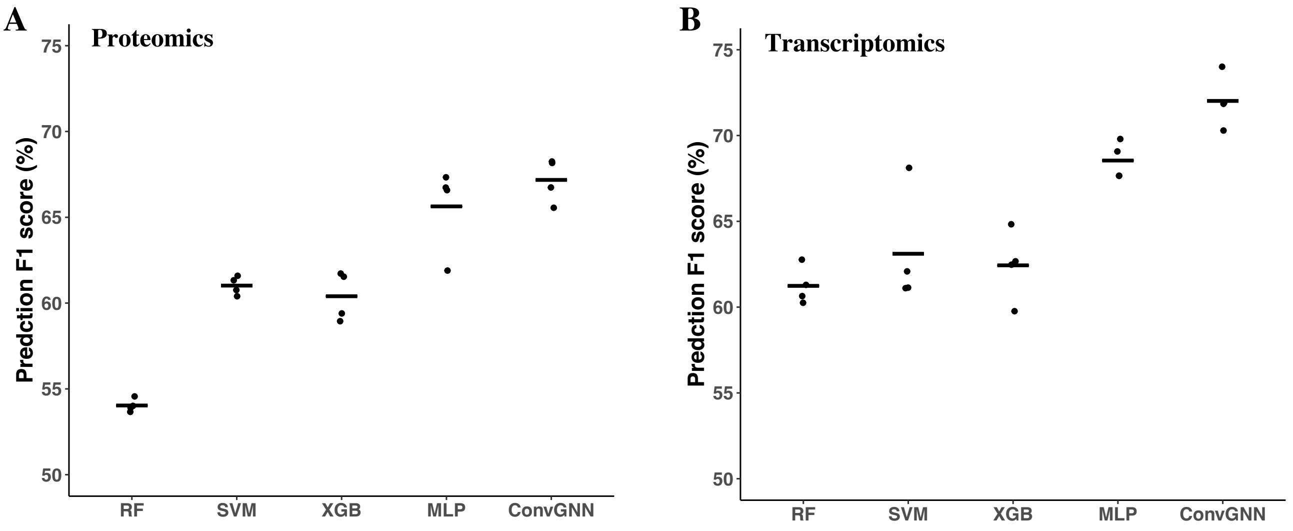

Figure S4: F1 score comparison between ConvGNN method and the other 4 approaches on single omics data. The ConvGNN models were trained in a 4-fold CV strategy with single omics data: proteomics data (A) or transcriptomics data (B). The STRING PPI network was used for the graph convolution. Four other classification methods were also evaluated: RF, SVM, XGB, and MLP. The model performances are assessed using the prediction F1 scores on the testing dataset. The lines represent the mean F1 scores for CV-trained models and the error bars represent the standard error of the mean.

Figure S5. Venn diagram of top 30 important features of ConvGNN and the other 4 methods.

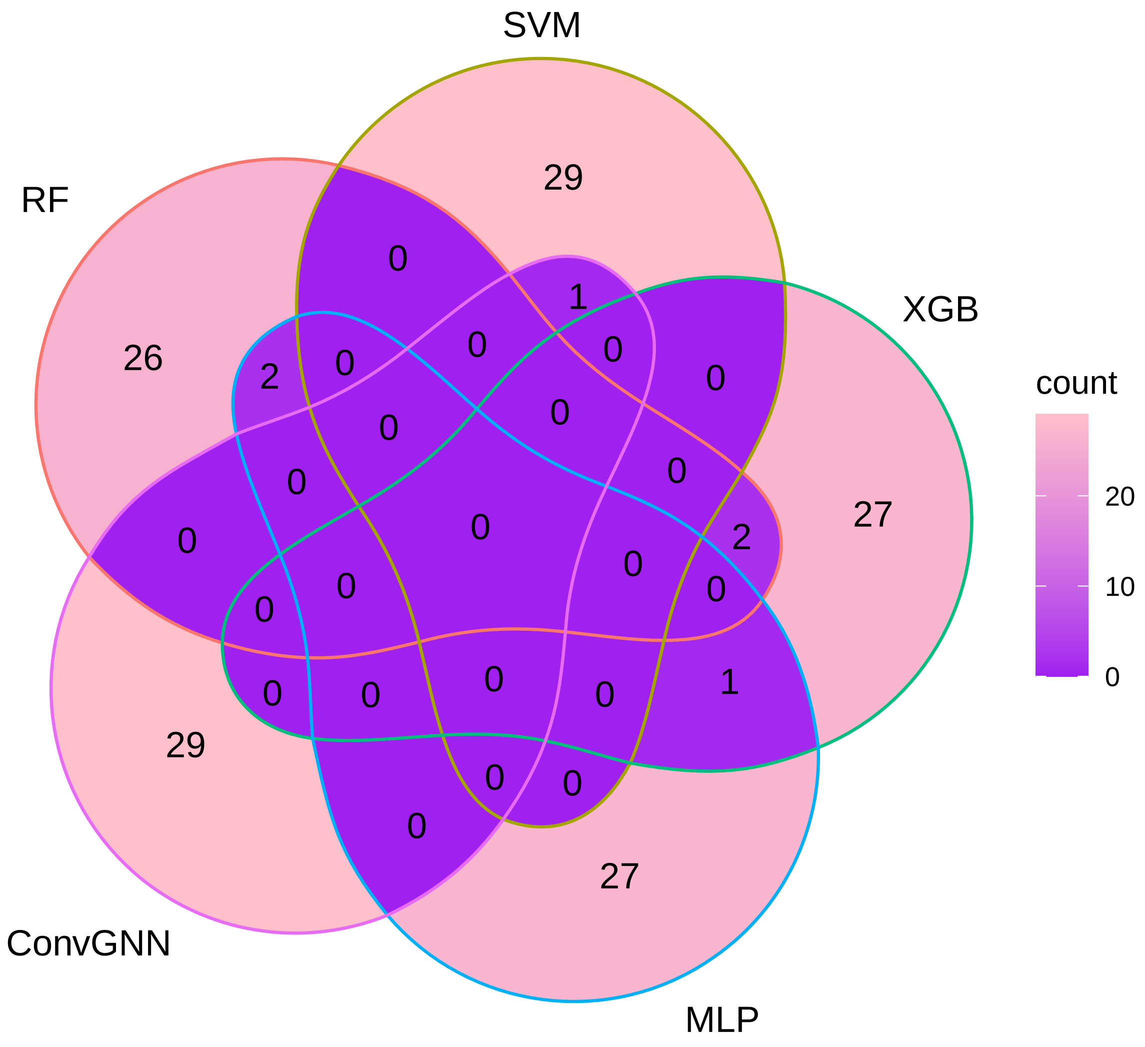

Figure S5: Venn diagram of top 30 important features of ConvGNN and the other 4 methods on two omics data. Top 30 important features were identified based on the SHAP values for ConvGNN, RF, SVM, XGB, and MLP.

3 SUPPLEMENTARY ACKNOWLEDGE

COPDGene Phase 3

Grant Support and Disclaimer

The project described was supported by Award Number U01 HL089897 and Award Number U01 HL089856 from the National Heart, Lung, and Blood Institute. The content is solely the responsibility of the authors and does not necessarily represent the official views of the National Heart, Lung, and Blood Institute or the National Institutes of Health.

COPD Foundation Funding

COPDGene is also supported by the COPD Foundation through contributions made to an Industry Advisory Board that has included AstraZeneca, Bayer Pharmaceuticals, Boehringer-Ingelheim, Genentech, GlaxoSmithKline, Novartis, Pfizer, and Sunovion.

COPDGene® Investigators – Core Units

Administrative Center: James D. Crapo, MD (PI); Edwin K. Silverman, MD, PhD (PI); Barry J. Make, MD; Elizabeth A. Regan, MD, PhD

Genetic Analysis Center: Terri H. Beaty, PhD; Peter J. Castaldi, MD, MSc; Michael H. Cho, MD, MPH; Dawn L. DeMeo, MD, MPH; Adel El Boueiz, MD, MMSc; Marilyn G. Foreman, MD, MS; Auyon Ghosh, MD; Lystra P. Hayden, MD, MMSc; Craig P. Hersh, MD, MPH; Jacqueline Hetmanski, MS; Brian D. Hobbs, MD, MMSc; John E. Hokanson, MPH, PhD; Wonji Kim, PhD; Nan Laird, PhD; Christoph Lange, PhD; Sharon M. Lutz, PhD; Merry-Lynn McDonald, PhD; Dmitry Prokopenko, PhD; Matthew Moll, MD, MPH; Jarrett Morrow, PhD; Dandi Qiao, PhD; Elizabeth A. Regan, MD, PhD; Aabida Saferali, PhD; Phuwanat Sakornsakolpat, MD; Edwin K. Silverman, MD, PhD; Emily S. Wan, MD; Jeong Yun, MD, MPH

Imaging Center: Juan Pablo Centeno; Jean-Paul Charbonnier, PhD; Harvey O. Coxson, PhD; Craig J. Galban, PhD; MeiLan K. Han, MD, MS; Eric A. Hoffman, Stephen Humphries, PhD; Francine L. Jacobson, MD, MPH; Philip F. Judy, PhD; Ella A. Kazerooni, MD; Alex Kluiber; David A. Lynch, MB; Pietro Nardelli, PhD; John D. Newell, Jr., MD; Aleena Notary; Andrea Oh, MD; Elizabeth A. Regan, MD, PhD; James C. Ross, PhD; Raul San Jose Estepar, PhD; Joyce Schroeder, MD; Jered Sieren; Berend C. Stoel, PhD; Juerg Tschirren, PhD; Edwin Van Beek, MD, PhD; Bram van Ginneken, PhD; Eva van Rikxoort, PhD; Gonzalo Vegas Sanchez-Ferrero, PhD; Lucas Veitel; George R. Washko, MD; Carla G. Wilson, MS;

PFT QA Center, Salt Lake City, UT: Robert Jensen, PhD

Data Coordinating Center and Biostatistics, *National Jewish Health, Denver, CO*: Douglas Everett, PhD; Jim Crooks, PhD; Katherine Pratte, PhD; Matt Strand, PhD; Carla G. Wilson, MS

Epidemiology Core, *University of Colorado Anschutz Medical Campus, Aurora, CO*: John E. Hokanson, MPH, PhD; Erin Austin, PhD; Gregory Kinney, MPH, PhD; Sharon M. Lutz, PhD; Kendra A. Young, PhD

Version Date: March 26, 2021

Mortality Adjudication Core: Surya P. Bhatt, MD; Jessica Bon, MD; Alejandro A. Diaz, MD, MPH; MeiLan K. Han, MD, MS; Barry Make, MD; Susan Murray, ScD; Elizabeth Regan, MD; Xavier Soler, MD; Carla G. Wilson, MS

Biomarker Core: Russell P. Bowler, MD, PhD; Katerina Kechris, PhD; Farnoush Banaei-Kashani, PhD

COPDGene® Investigators – Clinical Centers

Ann Arbor VA: Jeffrey L. Curtis, MD; Perry G. Pernicano, MD

Baylor College of Medicine, Houston, TX: Nicola Hanania, MD, MS; Mustafa Atik, MD; Aladin Boriek, PhD; Kalpatha Guntupalli, MD; Elizabeth Guy, MD; Amit Parulekar, MD;

Brigham and Women's Hospital, Boston, MA: Dawn L. DeMeo, MD, MPH; Craig Hersh, MD, MPH; Francine L. Jacobson, MD, MPH; George Washko, MD

Columbia University, New York, NY: R. Graham Barr, MD, DrPH; John Austin, MD; Belinda D'Souza, MD; Byron Thomashow, MD

Duke University Medical Center, Durham, NC: Neil MacIntyre, Jr., MD; H. Page McAdams, MD; Lacey Washington, MD

HealthPartners Research Institute, Minneapolis, MN: Charlene McEvoy, MD, MPH; Joseph Tashjian, MD

Johns Hopkins University, Baltimore, MD: Robert Wise, MD; Robert Brown, MD; Nadia N. Hansel, MD, MPH; Karen Horton, MD; Allison Lambert, MD, MHS; Nirupama Putcha, MD, MHS

Lundquist Institute for Biomedical Innovation at Harbor UCLA Medical Center, Torrance, CA: Richard Casaburi, PhD, MD; Alessandra Adami, PhD; Matthew Budoff, MD; Hans Fischer, MD; Janos Porszasz, MD, PhD; Harry Rossiter, PhD; William Stringer, MD

Michael E. DeBakey VAMC, Houston, *TX*: Amir Sharafkhaneh, MD, PhD; Charlie Lan, DO

Minneapolis VA: Christine Wendt, MD; Brian Bell, MD; Ken M. Kunisaki, MD, MS

Morehouse School of Medicine, Atlanta, GA: Eric L. Flenaugh, MD; Hirut Gebrekristos, PhD; Mario Ponce, MD; Silanath Terpenning, MD; Gloria Westney, MD, MS

National Jewish Health, Denver, CO: Russell Bowler, MD, PhD; David A. Lynch, MB

Reliant Medical Group, Worcester, MA: Richard Rosiello, MD; David Pace, MD

Temple University, Philadelphia, PA: Gerard Criner, MD; David Ciccolella, MD; Francis Cordova, MD; Chandra Dass, MD; Gilbert D'Alonzo, DO; Parag Desai, MD; Michael Jacobs, PharmD; Steven Kelsen, MD, PhD; Victor Kim, MD; A. James Mamary, MD; Nathaniel

Version Date: March 26, 2021

Marchetti, DO; Aditi Satti, MD; Kartik Shenoy, MD; Robert M. Steiner, MD; Alex Swift, MD; Irene Swift, MD; Maria Elena Vega-Sanchez, MD

University of Alabama, Birmingham, AL: Mark Dransfield, MD; William Bailey, MD; Surya P. Bhatt, MD; Anand Iyer, MD; Hrudaya Nath, MD; J. Michael Wells, MD

University of California, San Diego, CA: Douglas Conrad, MD; Xavier Soler, MD, PhD; Andrew Yen, MD

University of Iowa, Iowa City, IA: Alejandro P. Comellas, MD; Karin F. Hoth, PhD; John Newell, Jr., MD; Brad Thompson, MD

University of Michigan, Ann Arbor, MI: MeiLan K. Han, MD MS; Ella Kazerooni, MD MS; Wassim Labaki, MD MS; Craig Galban, PhD; Dharshan Vummidi, MD

University of Minnesota, Minneapolis, MN: Joanne Billings, MD; Abbie Begnaud, MD; Tadashi Allen, MD

University of Pittsburgh, Pittsburgh, PA: Frank Sciurba, MD; Jessica Bon, MD; Divay Chandra, MD, MSc; Joel Weissfeld, MD, MPH

University of Texas Health, San Antonio, San Antonio, TX: Antonio Anzueto, MD; Sandra Adams, MD; Diego Maselli-Caceres, MD; Mario E. Ruiz, MD; Harjinder Singh

Version Date: March 26, 2021
